# Increased risk of new-onset diabetes in patients with COVID-19: a systematic review and meta-analysis

**DOI:** 10.3389/fpubh.2023.1170156

**Published:** 2023-05-25

**Authors:** Jiajun Li, Yapeng Li, Zhenzhen Wang, Nanyang Liu, Lanye He, Han Zhang

**Affiliations:** ^1^Guang'anmen Hospital, China Academy of Chinese Medical Sciences, Beijing, China; ^2^Rehabilitation Therapy Center, Luoyang Orthopedic Hospital of Henan Province, Orthopedic Hospital of Henan Province, Luoyang, China; ^3^Department of Orthopaedic Surgery, Luoyang First People's Hospital, Luoyang, China; ^4^Department of Geratology, Xiyuan Hospital, China Academy of Chinese Medical Sciences, Beijing, China

**Keywords:** COVID-19, severe acute respiratory syndrome coronavirus 2 (SARS-CoV-2), new-onset, secondary diabetes, hyperglycemia

## Abstract

**Background:**

There is growing evidence that patients with COVID-19 are at increased risk of new-onset diabetes. The limited preliminary studies do not provide strong evidence. To assess the association of the SARS-CoV-2 virus with new-onset diabetes and to characterize the population.

**Methods:**

Search PubMed, Embase, Cochrane Library, and Web of Science electronic databases for a limited period from December 2019 to July 2022. Two independent reviewers conducted a thorough review of eligible articles and extracted relevant information. Pooled proportions, risk ratios (RR), and 95% confidence intervals (95% CI) indicated the incidence and risk ratios of events.

**Results:**

The incidence of new-onset diabetes and hyperglycemia in patients with COVID-19 was 5% (*P* < 0.001) (3 and 30% for new-onset diabetes and hyperglycemia, respectively), with age, ethnicity, time of diagnosis, and study type all having an impact on the incidence (*P* < 0.05). New-onset diabetes and hyperglycemia were 1.75 times higher in COVID-19 patients than in non-COVID-19 patients. In new-onset diabetes and hyperglycemia population, the percentage of men is 60% (40% for women), with a mortality rate of 17%. The proportion of new-onset diabetes and hyperglycemia after infection with COVID-19 was 25% in men and 14% in women.

**Conclusions:**

The incidence and relative risk of new-onset diabetes and hyperglycemia are elevated after COVID-19 infection, especially in the early COVID-19 and male populations.

**Systemic review registration:**

PROSPERO registration no.: CRD42022382989 https://www.crd.york.ac.uk/PROSPERO/display_record.php?RecordID=382989.

## Introduction

Since the outbreak of the epidemic at the end of 2019, COVID-19 is still spreading globally. As of November 2022, 629 million confirmed cases and 6.5 million deaths have been reported worldwide (due to the decline in confirmed cases worldwide, the actual number may be underestimated), which has a major impact on the global economy, society, and human health. The multiple factors of public health policy, viral mutations, and COVID-19 vaccine development and vaccination have resulted in a decline in new patients and mortality. While healthy people may be asymptomatic or recover within weeks of developing symptoms, they are also at risk for long-term COVID-19 disease (organ damage that makes it difficult to return to a healthy state, or increased risk of disease) (1).

Numerous investigations have demonstrated that the SARS-CoV-2 virus invades the respiratory system as well as a number of human tissues and organs, impairing their activities. In the study by Al-Aly et al. (2) the effects of COVID-19, which include neurological illnesses, mental health disorders, metabolic disorders, cardiovascular disease, gastrointestinal disorders, weariness, muscle-skeletal discomfort, and anemia, were thoroughly described. Similarly, it has been reported that the human pancreas is a target of the SARS-CoV-2 viral attack (3).

A growing number of clinical observations have shown that COVID-19 positive patients are at greater risk of developing diabetes than negative patients. One study highlighted that infection with the SARS-CoV-2 virus increases the risk of diabetes by ~40%, affecting ~2 in 100 patients (4). A recent meta-analysis by Zhang et al. (5) reported the risk of new-onset diabetes after COVID-19, but the study only included cohort studies. Moreover, this inconsistency also hinders our understanding of the causal relationship between them. As new data become available, there is a need to reassess the relationship between COVID-19 and new-onset diabetes. Therefore, more literature was included in this meta-analysis to assess the incidence of new-onset diabetes after COVID-19.

## Method

### Search strategy

The current systematic review and meta-analysis follow the Preferred Reporting Items for Systematic Reviews and Meta-Analyses (PRISMA) (Appendix S1) (6) and the study is registered with PROSPERO (Registration number: CRD42022382989). Two independent reviewers (JJL and YPL) systematically searched electronic databases PubMed, Embase, Cochrane Library, and Web of Science for relevant literature from December 2019 to July 2022. The search strategy combined three constructs: COVID-19 or SARS-CoV-2, diabetes or hyperglycemia, and new start or second or new diagnosis. We searched for relevant literature regardless of country, language, or article type. See Appendix S2 for detailed search strategies.

### Inclusion and exclusion criteria

The inclusion criteria of this study followed the PICOS framework: (1) Participant (P): non-diabetic patients or blood glucose levels within the normal range; (2) Intervention (I): infected with COVID-19; (3) Comparison (C): matched to the experimental group and not infected with COVID-19 (case-control study) or no control group (cross-sectional study); (4) Outcome (O): the number of people with new-onset diabetes or blood glucose values exceeding the normal range; (5) Study design (S): cross-sectional studies, cohort studies, or case-control studies. Data given in the relevant literature were excluded if they could not be extracted or the full text could not be found.

### Data extraction and literature quality assessment

Based on the above inclusion and exclusion criteria, two independent reviewers (JJL and YPL) conducted a thorough review of eligible articles and extracted the following information: author, year, country, type of study, participants (sample size, age, number of males [calculated when not directly stated], number of newly diagnosed diabetes), diagnostic criteria for diabetes, time of diabetes diagnosis, type of diabetes, and quality of literature. The quality of the literature for cross-sectional studies, cohort studies, and case-control studies was assessed using the Agency for Health care Research and Quality (AHRQ) (11 points total; low quality 0–3; moderate quality 4–7; high quality 8–11) (7) and the Newcastle-Ottawa Score (NOS) (nine stars; ≤ six stars for poor quality; seven and eight stars for moderate quality; nine stars for high quality) (8). If opinions diverged, they were resolved by consulting a third author.

### Statistical analysis

Statistical software R (version 4.1.1) was used for statistical analysis. We used the metaprop package to calculate the pooled risk ratio (RR) and 95% confidence intervals (95% CI). A random-effects model was selected if *I*^2^ ≥ 50% and a fixed-effects model was selected if the opposite was true. Subgroup analysis was used to explore potential sources of heterogeneity. Subsequently, the publication bias of the included literature was analyzed by Egger's and Begg's tests. We also constructed funnel plots as a visualization to assess the possibility of publication bias. Furthermore, we tested the robustness of the results by sequentially removing and accumulating each study.

## Result

### Search results and article selection

**Figure 1:**
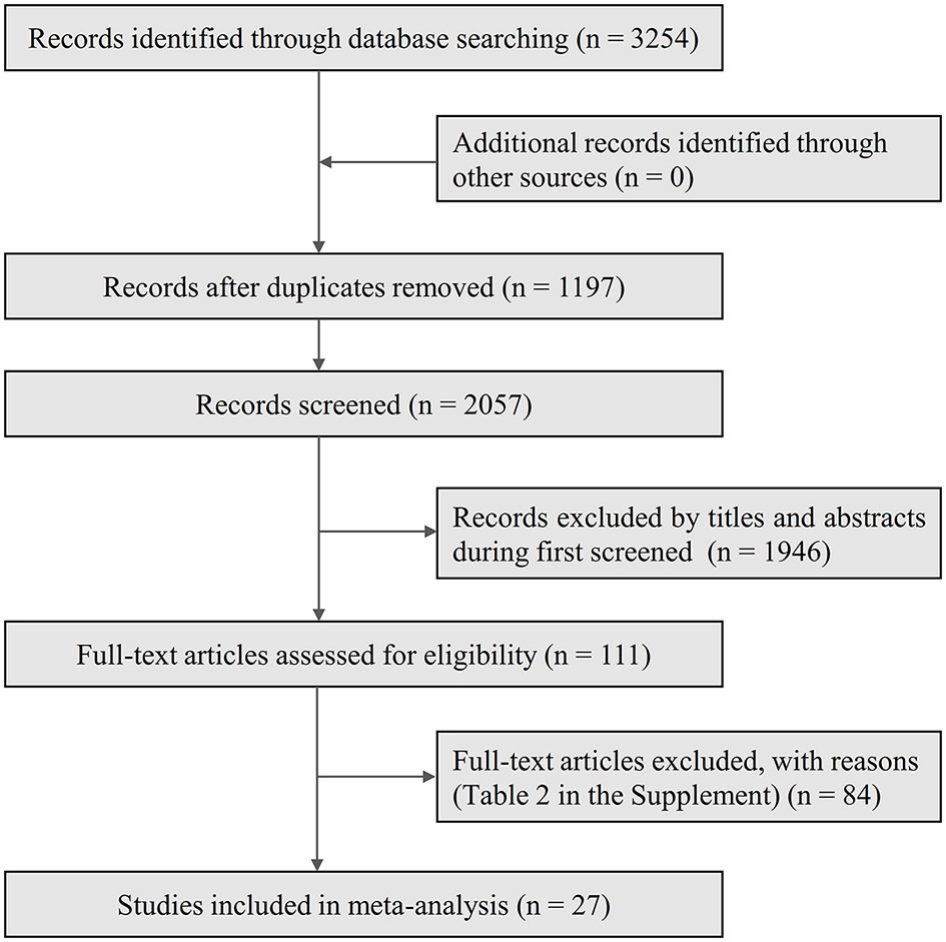
Flow chart of literature screening.

We retrieved a total of 3,254 relevant literature from the four databases, and there were still 2,057 pieces of literature after removing duplicate entries. Subsequent scans of titles and abstracts left 111 articles after removing reviews, conference abstracts, case reports, experimental registers, and studies whose content was not relevant to the purpose of this study. The remaining literature was further screened by reviewing the full text according to the inclusion and exclusion criteria formulated above, and 27 articles that met the criteria were eventually included, while 84 were excluded for reasons such as reviews, case reports, or irrelevance to the study content, or unavailability of data. The detailed literature search process is shown in Figure 1.

### Characteristics of the included studies

The 27 included studies included 14 cross-sectional studies, 11 cohort studies, and 2 case-control studies, including a total of 3,976,089 COVID-19 patients and 33,069,542 non-COVID-19 patients. Nine of the studies were from China (9–17), eight from the United States (4, 18–24), three each from India (25–27) and Italy (28–30), and one each from Bangladesh (31), Egypt (32), the United Kingdom (33), and South Africa (34). Sample sizes ranged from 66 to 2,489,266. The time of diagnosis of new-onset diabetes or hyperglycemia was either newly identified during hospitalization or within an established follow-up period. Four of the studies reported both new-onset diabetes and hyperglycemia (9, 11, 12, 30), three studies reported only new-onset hyperglycemia (16, 17, 25), while the remaining studies reported only new-onset diabetes. Notably, two studies were younger than 18 years (18, 21), four studies had no age limit (22, 26, 31, 33), and five studies did not report age (11, 14, 15, 27, 28), and the remaining 16 studies had ages ≥18 years. The basic characteristics of the included studies are summarized in Tables 1, 2.

**Table 1:** The main characteristics of the study of new-onset diabetes and hyperglycemia in the COVID-19 population.

**References**	**Country**	**Study type**	**Ethnicity**	**Study period**	**COVID-19**	**Age**	**Male, %**	**Event**	**Definition of NDD**	**Time of diagnosis**	**Type of diabetes**	**Study quality**
Akter et al. (31)	Bangladesh	Cross-sectional study	Asian	April 1 and June 30, 2020	598	No restrictions	NR	10	HbA1c ≥ 6.5% or a random glucose level ≥ 11.1 mmol/l	During hospitalization	NR	3
Cromer et al. (20)	USA	Cross-sectional study	Caucasian	March and September 2020	1,385	≥18	NR	77	No prior history of diabetes, HbA1c ≥ 6.5%, or 2 International Disease Classification codes for any form of diabetes, insulin use, or severe hyperglycemia (≥16.7 mmol/L) at admission	During hospitalization	T2D: 62; The rest did not report	5
Fadini et al. (28)	Italy	Cross-sectional study	Caucasian	February and April 2020	327	Unclear	189 (57.80%)	21	HbA1c ≥ 6.5% or a random glucose level ≥ 11.1 mmol/l, accompanied by signs and symptoms of hyperglycemia	During hospitalization	NR	4
Farag et al. (32)	Egypt	Cross-sectional study	Caucasian	1 April 2020 to 31 May 2020	558	≥18	310 (55.56%)	65	No preceding history of DM with FPG ≥ 126 mg/dL or RBG ≥ 200 mg/dL and HbA1c <6.5% or previously undiagnosed DM (FPG ≥ 126 mg/dL or RBG ≥ 200 mg/dL and HbA1c ≥ 6.5% or HbA1c ≥ 6.5% only)	During hospitalization	T1D: 7; T2D:58	4
Lampasona et al. (29)	Italy	Cross-sectional study	Caucasian	25 February and 19 April 2020	419	≥18	272 (64.92%)	49	If patients without a diagnosis of diabetes had a mean FPG ≥ 7.0 mmol/l during the hospitalization for COVID-19 pneumonia	During hospitalization	NR	4
Li et al. (9)	China	Case-control study	Asian	January 22, 2020 to March 17, 2020	355	≥18	180 (50.70%)	NDD: 94 NOH: 129 Total: 223	Hyperglycaemia: fasting glucose 5.6–6.9 mmol/L and/or HbA1c 5.7–6.4%; NDD: fasting glucose ≥ 7 mmol/L and/or HbA1c ≥ 6.5%	During hospitalization	NR	6
Lin et al. (10)	China	Cohort study	Asian	December 30th, 2019 and April 12th, 2020	3,114	18–75	1,390 (44.64%)	351	At least two FPG readings ≥ 7 mmol/L	During hospitalization	NR	6
Mithal et al. (25)	India	Cross-sectional study	Asian	July 9, 2020, to August 8, 2020	212	18–92	135 (63.68%)	NOH: 21	Those who did not meet the criteria for diabetes but required insulin to maintain normoglycemia were classified as NOH	During hospitalization	NR	4
Nesan et al. (26)	India	Cross-sectional study	Asian	June to November 2020	1,222	No restrictions	NR	10	HbA1c ≥ 6.5%	After discharge	NR	3
Rajueni et al. (27)	India	Cross-sectional study	Asian	December 2020 to April 2021	629	Unclear	NR	12	HbA1c ≥ 6.5%	After discharge	NR	3
Smith et al. (23)	USA	Cross-sectional study	Caucasian	16 March-2 May 2020	70	21–100	NR	29	NDD was defined by persistently elevated FBG > 125 mg/dL and requiring insulin therapy.	During hospitalization	NR	4
Van der Westhuizen et al. (34)	South Africa	Cross-sectional study	Caucasian	8 June 2020–18 August 2020	897	≥18	NR	125	HbA1c ≥ 6.5%	During hospitalization	NR	4
Wang S. et al. (11)	China	Cohort study	Asian	24 January 2020 to 10 February 2020	605	Unclear	322 (53.22%)	NDD: 176 NOH: 100 Total: 276	According to WHO guidelines in terms of admission FBG (<6.1, 6.1–6.9, and ≥7.0 mmol/l)	During hospitalization	NR	6
Wang Z. et al. (12)	China	Cross-sectional study	Asian	February 9–28, 2020	101	24–88	NR	NDD: 16 NOH: 44 Total: 60	WHO guidelines on medicines for diabetes treatment intensification	During hospitalization	NR	5
Yang et al. (13)	China	Cross-sectional study	Asian	January 29, 2020, to March 20, 2020	69	≥18	34 (49.28%)	21	FBG ≥ 7.0 mmol/L for two times during hospitalization, without glucocorticoid treatment, and without a history of diabetes in COVID-19 patients were defined as NDD	During hospitalization	NR	6
Yi et al. (14)	China	Cross-sectional study	Asian	January to February 2020	470	Unclear	NR	3	HbA1c ≥ 6.5%	During hospitalization	NR	3
Yuan et al. (15)	China	Cross-sectional study	Asian	10 January 2020 and 30 March 2020	740	Unclear	361 (48.78%)	187	HbA1c ≥ 6.5%	During hospitalization	NR	5
Zhang et al. (16)	China	Cohort study	Asian	February 8 to March 21, 2020	105	≥18	52 (49.52%)	NOH: 21	FPG levels of ≥7.0 mmol/L once and HbA1c levels of <6.5%	During hospitalization	NR	6
Zhou et al. (17)	China	Case-control study	Asian	January to March 2020	66	≥18	38 (57.58%)	NOH: 22	No past histories of diabetes, HbA1c <6.5%, RBG > 11.1 mmol/L during hospitalization, and normal blood glucose after discharge from the hospital	During hospitalization	NR	5

DM, diabetes mellitus; FBG, fasting blood glucose; FPG, fasting plasma glucose; HbA1c, hemoglobin A1c; NDD, Newly diagnosed diabetes; NOH, New onset hyperglycemia; NR, Not reported; RBG, random blood glucose; T1D, type 1 diabetes; T2D, type 2 diabetes; WHO, World health organization.

**Table 2:** The main characteristics of the study of new-onset diabetes and hyperglycemia in the COVID-19 population versus the non-COVID-19 population.

**References**	**Country**	**Study type**	**Ethnicity**	**Study period**	**COVID-19 patients**				**Non-COVID-19 patients**				**Definition of NDD**	**Time of diagnosis**	**Type of diabetes**	**Study quality**
					***N***	**Age**	**Male, %**	**Event**	***N***	**Age**	**Male, %**	**Event**				
Ayoubkhani et al. (33)	UK	Cohort study	Caucasian	1 January to 31 August 2020	36,100	No restrictions	NR	400	36,100	No restrictions	NR	125	Primary and secondary ICD-10 codes (codes U07.1 and U07.2)	After discharge	T1D or T2D	7
Barrett et al. (18) (HealthVerity)	USA	Cohort study	Caucasian	March 1, 2020–June 28, 2021	439,439	<18	219,427 (49.93%)	1,120	439,439	<18	219,427 (49.93%)	853	One or more health care claims with a diabetes diagnosis (ICD-10-CM codes E08–E13) occurring >30 days after the index date (excluding cases of transient, resolved hyperglycemia)	After discharge	T1D or T2D (94.0%)	6
Barrett et al. (18) (IQVIA)	USA	Cohort study	Caucasian	March 1, 2020–June 28, 2021	80,893	<18	40,376 (49.91%)	68	404,465	<18	201,880 (49.91%)	132	One or more health care claims with a diabetes diagnosis (ICD-10-CM codes E08–E13) occurring >30 days after the index date (excluding cases of transient, resolved hyperglycemia)	After discharge	T1D or T2D (94.1%)	6
					***N***	**Age**	**Male, %**	**Event**	***N***	**Age**	**Male, %**	**Event**				
Birabaharan et al. (19)	USA	Cohort study	Caucasian	20 January 2020 to 20 January 2021	324,360	≥18	NR	3,934	330,734	≥18	NR	2,632	One or more ICD-10 E11	After discharge	T2D	6
Kendall et al. (21)	USA	Cohort study	Caucasian	March 2020 and December 2021	285,628	<18	143,289 (50.17%)	123	285,628	<18	144,029 (50.43%)	72	ICD-10 code U07.1	After discharge	T1D	5
Laurenzi et al. (30)	Italy	Cohort study	Caucasian	February 25 to May 15, 2020	471	≥18	NR	NDD: 39 NOH: 256 Total: 295	64	≥18	NR	NDD: 7 NOH: 15 Total: 22	(1) They had a negative history of diabetes, no prescription of diabetes medications, and a FBG during hospitalization, in the absence of infusions of dextrose, of 7.0 mmol/L or higher (ADA criteria); (2) Hyperglycemia not in the diabetes range if they had random blood glucose levels between 100 and 199 mg/dL or 2 FBG >100 and <126 mg/dL;	During hospitalization	NR	4
Qeadan et al. (22)	USA	Cohort study	Caucasian	December 1, 2019 through July 31, 2021	2,489,266	No restrictions	1,081,608 (43.45%)	5,163	24,803,613	No restrictions	10,579,475 (42.65%)	36,348	T1D associated ICD-10 codes	During hospitalization	T1D	7
					***N***	**Age**	**Male, %**	**Event**	***N***	**Age**	**Male, %**	**Event**				
Wander et al. (24)	USA	Cohort study	Caucasian	1 March 2020 and 10 March 2021	126,710	≥18	109,693 (86.57%)	748	2,651,058	≥18	2,291,801 (86.45%)	8,402	(1) Two or more abnormal laboratory values from plasma or serum (random glucose ≥ 200 mg/dL, fasting glucose ≥ 126 mg/dL, 2-h glucose from an oral glucose tolerance test ≥ 200 mg/dL) or whole blood (A1c ≥ 6.5%); (2) Two outpatient or one inpatient ICD-10 codes of E08–E13; or (3) receipt of an initial and one refill prescription of a glucose-lowering medication.	After discharge	T1D, T2D or other	7
Xie et al. (4)	USA	Cohort study	Caucasian	March 1, 2020, and Sept 30, 2021	181,280	≥18	159,666 (88.08%)	7,396	4,118,441	≥18	3,655,034 (88.75%)	127,858	The ICD-10 codes (E08.X to E13.X) or a HbA1c measurement of more than 6.4% (46 mmol/mol)	After discharge	Mostly T2D	7

ADA, American Diabetes Association; FBG, fasting blood glucose; HbA1c, hemoglobin A1c; ICD-10, the International Classification of Diseases, 10th revision; NDD, Newly diagnosed diabetes; NOH, New onset hyperglycemia; NR, Not reported; T1D, type 1 diabetes; T2D, type 2 diabetes.

### Quality assessment

The AHRQ was used to assess the quality of the literature for cross-sectional studies, of which four were rated as low quality (14, 26, 27, 31) and the remaining 10 studies were rated as moderate quality. The mean score was 4.07 and was judged to be of moderate quality overall. NOS was used to assess the quality of the literature for both cohort and case-control studies. Four of the cohort studies were rated as moderate quality (4, 22, 24, 33), and the remaining seven were rated as low quality. The average score was 6.1, and the comprehensive quality is medium. Both case-control studies were of low quality, with an average score of 5.5 (Tables 1, 2). Subgroup analysis based on the quality level of the literature showed a higher proportion of new-onset diabetes and hyperglycemia in the medium-quality literature (RR = 0.07; 95% CI, 0.03–0.17). However, there was no significant difference between the two groups (*P* = 0.27) (Table 3).

**Table 3:** Subgroup analysis of new-onset diabetes and hyperglycemia.

	**No. of studies**	**Proportion/RR**	**95%CI**	***P***	***I* ^2^**	***Q***	***P*_subgroup_(*X^2^ ^*test*^*)**
**Incidence of new-onset diabetes and hyperglycemia in the COVID-19 population**							
**Age**							
No restrictions	4	0.01	0.00–0.02	<0.01	100%	70.27	<0.01
<18	3	0.00	0.00–0.00	<0.01	100%		
≥18	16	0.13	0.07–0.25	0	100%		
Unclear	5	0.07	0.01–0.30	<0.01	99%		
**Ethnicity**							
Asian	13	0.11	0.04–0.26	<0.01	99%	4.83	0.03
Caucasian	15	0.02	0.01–0.07	0	100%		
**Time of diagnosis**							
During hospitalization	18	0.13	0.06–0.27	0	100%	22.14	<0.01
After discharge	10	0.01	0.00–0.02	0	100%		
**Study type**							
Cross-sectional study	14	0.08	0.04–0.16	<0.01	98%	25.35	<0.01
Cohort study	12	0.02	0.00–0.07	0	100%		
Case-control study	2	0.47	0.25–0.87	<0.01	92%		
**Study quality**							
Low	14	0.03	0.01–0.11	0	100%	1.24	0.27
Moderate	14	0.07	0.03–0.17	0	100%		
**Sample size**							
<10,000	20	0.14	0.07–0.25	0	99%	35.54	<0.01
>10,000	8	0.00	0.00–0.01	0	100%		
**The risk ratio for new-onset diabetes and hyperglycemia in COVID-19 vs. non-COVID-19 populations**							
**Age**							
No restrictions	2	2.11	0.95–4.70	<0.01	98%	0.68	0.71
<18	3	1.76	1.19–2.60	<0.01	90%		
≥18	4	1.58	1.33–1.88	<0.01	97%		
**Time of diagnosis**							
During hospitalization	2	1.51	1.22–1.88	0.15	51%	1.12	0.29
After discharge	7	1.81	1.41–2.32	<0.01	97%		
**Study quality**							
High	4	1.81	1.23–2.66	<0.01	98%	0.09	0.77
Moderate	5	1.69	1.36–2.11	<0.01	83%		

RR, risk ratio.

### Main results of the meta-analysis

#### Incidence of new-onset diabetes and hyperglycemia after COVID-19 infection

**Figure 2:**
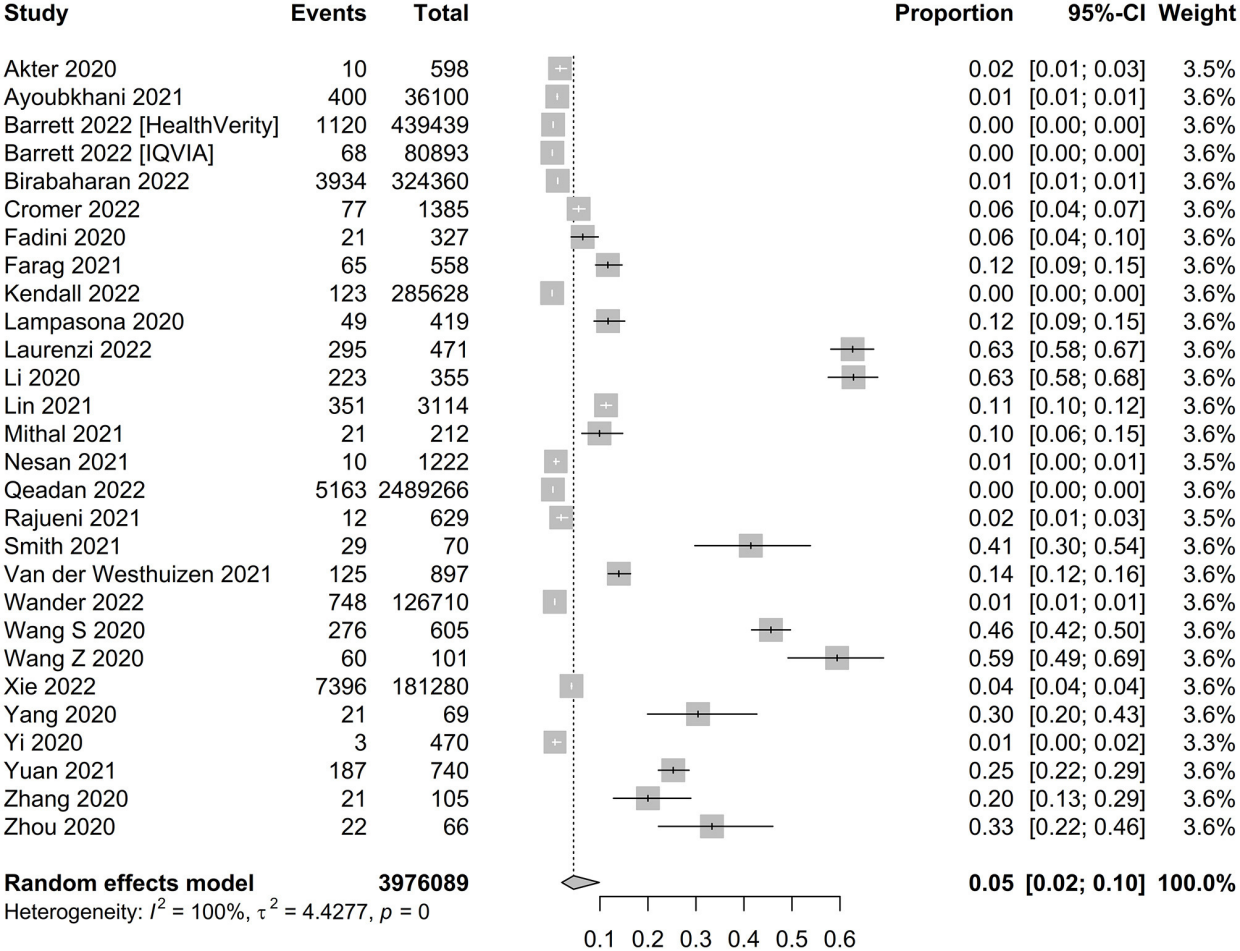
Forest plot of the incidence of new-onset diabetes and hyperglycemia.

The pooled proportion of new-onset diabetes and hyperglycemia after a positive diagnosis of COVID-19 was 0.05 (95% CI, 0.02–0.10, *P* < 0.001, *n* = 3,976,089) (Figure 2). Age subgroup analysis showed a higher proportion of new-onset diabetes and hyperglycemia in patients aged ≥ 18 years compared with those aged <18 years (0.13 for ≥18 years old, 0.00 for <18 years old, *P* < 0.01). In a subgroup analysis by race, the prevalence of new-onset diabetes and hyperglycemia was significantly higher in Asian than in Caucasian populations (0.11 for Asian, 0.02 for Caucasian; *P* = 0.03). According to the subgroup analysis of diagnosis time, the proportion of new-onset diabetes and hyperglycemia diagnosed during hospitalization was significantly higher than after discharge (0.13 during hospitalization, 0.01 after discharge, *P* < 0.01). Likewise, subgroup analysis by study type showed the highest proportion of pooled case-control studies (0.47 for case-control studies, 0.08 for cross-sectional studies, and 0.02 for cohort studies, *P* < 0.01). In addition, subgroup analysis of study quality showed a higher proportion of studies combined with medium quality scores than with low quality scores, however, there was no significant difference between groups (0.07 for medium quality and 0.03 for low quality; *P* = 0.27). Analysis based on sample size subgroups showed that the prevalence of new-onset diabetes and hyperglycemia was significantly higher in studies with small sample sizes than in studies with large sample sizes (0.14 for sample sizes <10,000 and 0.00 for sample sizes >10,000; *P* < 0.01). We then performed a meta-analysis of new-onset diabetes and new-onset hyperglycemia, which showed that the proportion of new-onset diabetes was 0.03 (95% CI, 0.01–0.07, *P* = 0, *n* = 3,975,706) (Supplementary Figure S1), and the proportion of new-onset hyperglycemia was 0.30 (95% CI, 0.18–0.42, *P* < 0.01, *n* = 1,915) (Supplementary Figure S2).

#### New-onset diabetes and hyperglycemia in COVID-19 patients vs. non-COVID-19

Overall, the pooled RR values based on a randomized controlled model showed a significant difference in the proportion of COVID-19 (*n* = 3,964,147) and non-COVID-19 (*n* = 33,069,542) groups with new-onset diabetes and hyperglycemia (RR = 1.75, 95% CI, 1.43 to 2.14) and significant heterogeneity (*I*^2^ = 96%; *P* < 0.01) (Supplementary Figure S3). Subgroup analysis of age, study quality, and time of diagnosis all showed no significant differences between subgroups (all *P* > 0.05) (Table 3).

#### Characteristics of new-onset diabetes and hyperglycemia populations in COVID-19 patients

Notably, there were more men than women in the new-onset diabetes and hyperglycemia groups (60 and 40%, respectively, *P* < 0.01, *n* = 6,182) (Supplementary Figures S4, S5) Mortality in the new-onset diabetes and hyperglycemia group was 17% (95% CI, 0.11–0.25, *P* < 0.01, *n* = 1,161) (Supplementary Figure S6). In another analysis, men had higher rates of new-onset diabetes and hyperglycemia after COVID-19 infection than women (men: 25%, 95% CI, 0.11–0.38, *P* < 0.01, *n* = 1,084,297; women: 14%, 95% CI, 0.06–0.24, *P* < 0.01, *n* = 1,266,926) (Supplementary Figures S7, S8).

### Sensitivity analysis

We used a removal-by-study and cumulative-by-study approach to evaluate the stability of the results of the above two meta-analyses. First, under the random-effects model, we sequentially deleted one literature and merged the remaining literature, and the results showed that none of the merged values were significantly different after deleting a study (Supplementary Figures S9, S10). Similarly, for the cumulative method, under the random-effects model, we used sequentially adding one literature and merging the results, and the results showed that none of the merged values were significantly different after adding a study (Supplementary Figures S11, S12) In conclusion, both methods showed robust results.

### Publication bias

We performed a publication bias test for meta-analysis of the incidence of new-onset diabetes and hyperglycemia after infection with COVID-19 (*n* > 10). We found no publication bias by Egger test (intercept = −4.4375, SE = 0.4392, *P* = 0.1971). (Supplementary Figure S13A) Similarly, Begg's test results showed no publication bias (*P* = 0.1332) (Supplementary Figure S13B). Visual review of funnel plots revealed publication bias (Supplementary Figure S13C), and we used the cut-and-patch method to add nine studies to achieve symmetry (Supplementary Figure S13D).

## Discussion

To the best of our knowledge, this study is the most recent meta-analysis evaluating the occurrence of new-onset diabetes and hyperglycemia after infection with COVID-19. Overall, the incidence of new-onset diabetes and hyperglycemia after COVID-19 infection was 5%, with age, ethnicity, time of diagnosis, and study type all having an impact on the incidence. Notably, the incidence was 3 and 30% when new-onset diabetes and new-onset hyperglycemia were proportionally combined separately. Further, new-onset diabetes and hyperglycemia were 1.75 times higher in COVID-19 patients than in non-COVID-19 patients. Statistical description of the population with new-onset diabetes and hyperglycemia revealed a higher proportion of males (60%) than females (40%) and a mortality rate of 17%. Based on the total sample size, a much higher proportion of male COVID-19 patients (25%) developed new-onset diabetes and hyperglycemia than females (14%).

According to the latest data released by the International Diabetes Federation, there are 537 million people with diabetes worldwide. This meta-analysis found an alarming 5% prevalence of new-onset diabetes and hyperglycemia after COVID-19 infection in people without prior diabetes. Even more worrisome is the reported higher risk of death from new-onset diabetes compared to known diabetes in patients hospitalized with COVID-19 (9). In a recent study on the association between COVID-19 and diabetes in children (under 18 years of age), an almost 72% increase in newly diagnosed T1D was seen in patients with COVID-19 compared to the non-COVID-19 respiratory population (21). However, the prevalence of new-onset diabetes and hyperglycemia was much lower in children than in adults in the age-based subgroup analysis. This result may be related to the fact that fewer studies are currently examining COVID-19 and childhood diabetes. Subgroup analysis for ethnicity showed an incidence of 11% in Asian populations and 2% in Caucasians, a result that may be related to genetic susceptibility (35). Further, we combined the incidence of new-onset diabetes and hyperglycemia during hospitalization and post-discharge separately, showing a 13% incidence in hospital and only a 1% incidence after discharge. This interesting phenomenon may be observed in the study by Kendall et al. (21), where there was a decreasing trend in the number of new T1D diagnoses at 1, 3, and 6 months, respectively. More interestingly, Cromer et al. reported that newly diagnosed diabetes was characterized by hospitalized hyperglycemia that usually subsided after the acute illness subsided, with the final data showing that only 7.8% of patients required insulin (20). Subgroup analysis by study type showed a high incidence of new-onset diabetes and hyperglycemia in the case-control group of 47%, which we interpreted as perhaps an error due to the small sample size. Similarly, subgroup analyses by study quality, which we attributed to the small number of low- and high-quality included literature, had a larger error. Interestingly, for subgroup analysis by sample size, we found that the small sample study prevalence (14%) was much higher than the large sample study prevalence (0.00%). We speculate that the possible reason for this is that the large sample data were derived from a web-based database, whereas the small sample studies were mostly from inpatients. In contrast, hospitalized patients either had the comorbid underlying disease or severe clinical symptoms after SARS-CoV-2 infection. When we combined the incidence of new-onset diabetes and new-onset hyperglycemia separately, we found that the incidence of new-onset hyperglycemia (30%) was much higher than that of new-onset diabetes (3%), which we explain as a possible reason for this phenomenon because the time of diagnosis in all studies of new-onset hyperglycemia was during hospitalization.

The results of this meta-analysis showed that the risk of new-onset diabetes and hyperglycemia was significantly higher in the COVID-19 population than in the non-COVID-19 population (RR = 1.75). The current study suggested a bidirectional relationship between COVID-19 and diabetes (36). In other words, COVID-19 not only aggravates the condition in diabetic patients, but also may induce new-onset diabetes in normal individuals. Cromer et al. (20) suggested that COVID-19 infection may not directly cause diabetes, but may promote further progression in patients with prediabetes or in those with undiagnosed diabetes; the authors also suggested that new-onset diabetes and hyperglycemia may be a transitional disease related to COVID-19. In conclusion, we suggest that the significantly higher incidence of new-onset diabetes and hyperglycemia in the COVID-19 population compared with the non-COVID-19 population may be the result of the combined effect of “a bidirectional relationship between COVID-19 and diabetes”.

The current meta-analysis found a higher proportion of men than women with new-onset diabetes and hyperglycemia (60 vs. 40%). Based on the total sample size, the probability of new-onset diabetes and hyperglycemia after infection with COVID-19 was similarly higher in men than in women (25 vs. 14%). According to the International Diabetes Federation 2021 Global Diabetes Map (10th edition), the prevalence of diabetes was slightly higher in men than in women among adults aged 20–79 years (10.8 vs. 10.2%). Therefore, we suggested that males are more likely to develop new-onset diabetes when SARS-CoV-2 virus induces new-onset diabetes in healthy population compared to females. Further, it has been suggested that the interaction between COVID-19 and diabetes increases serum inflammatory cytokine levels as an important cause of death in heavy and critically ill COVID-19 patients (37).

Currently, some scholars believed that the new-onset diabetes caused by SARS-CoV-2 might be a new type of diabetes (36) and perhaps a transient hyperglycemia (20). Laboratory studies have shown that pancreatic islet cells are highly sensitive to SARS-CoV-2 virus. SARS-CoV-2 infection causes islet cell stress response and high expression of chemokines (3). Wu et al. (38) found that SARS-CoV-2 receptors (ACE2 and related entry factors, such as TMPRSS2, NRP1, and TRFC) were expressed in β-cells after infection with the virus, which in turn attenuated insulin expression levels and induced β-cell apoptosis. However, Accili (39) argued that diabetic ketoacidosis after COVID-19 should require conventional insulin therapy if the virus causes permanent loss of β-cell function, but the challenge in clinical practice is mainly extrinsic to the β-cells. In conclusion, the mechanism between COVID-19 and diabetes mellitus needs to be further demonstrated.

There are some limitations of this study. First, most of the studies lacked baseline data prior to COVID-19 infection. According to the theory of the “bidirectional relationship between COVID-19 and diabetes”, new-onset diabetes and hyperglycemia may be the result of pre-diabetes or further progression in patients with undiagnosed diabetes. Second, according to Cromer et al. (20), who observed that patients with COVID-19 have “early onset hyperglycemia and a large part of it subsides later”, the incidence of new-onset diabetes and hyperglycemia varies considerably by the time of diagnosis. Therefore, the inconsistent follow-up time after discharge had a degree of influence on the judgment of the outcome. Third, corticosteroids (glucocorticoids) as a routine drug for the treatment of patients with COVID-19 also caused hyperglycemia in patients (40), and we could not determine whether new-onset diabetes and hyperglycemia were caused by SARS-CoV-2 or corticosteroids. Finally, risk factors for new-onset diabetes (age, obesity, pregnancy, mental status and family history of diabetes, etc.) were not assessed in groups, which does not facilitate a precise understanding of COVID-19-induced new-onset diabetes and hyperglycemia. In conclusion, our knowledge of SARS-CoV-2 is still limited.

## Conclusion

The incidence and relative risk of new-onset diabetes and hyperglycemia are elevated after COVID-19 infection, especially in the early COVID-19 and male populations. We hypothesize that COVID-19-related hyperglycemia may be a transient phenomenon, with most patients returning to normal blood glucose ranges over time. Future researchers should work on the potential mechanisms of the relationship between COVID-19 and diabetes to provide effective preventive measures and treatments for the development of diabetes in the context of COVID-19.

## Author contributions

JL, ZW, and YL participated in the literature searches, abstracts and full-text reviews, data extraction, synthesis and interpretation of data, and drafting of manuscripts. HZ and LH contributed to evaluating the quality of the literature studies and critically revised the manuscript. NL and LH initiated and designed the study, helped to explain the data, and modified the manuscript. All authors read and approved the final manuscript and are responsible for the content.
