# Supplementary material for: Increased risk of new-onset diabetes in patients with COVID-19: a systematic review and meta-analysis
Source: Front Public Health. 2023 May 25;11:1170156. doi: 10.3389/fpubh.2023.1170156 (PMC10248182; doi:10.3389/fpubh.2023.1170156)

**1. Search strategy**

**2. Excluded articles at full-text assessment (n=84)**

**3. Figure legends of the supplementary**

**1. Search strategy**

***PubMed***

((("COVID-19"[Mesh]) OR ((((((((((((((((((((((2019 nCoV Disease[Title/Abstract]) OR (2019 nCoV Infection[Title/Abstract])) OR (2019 Novel Coronavirus[Title/Abstract])) OR (2019 Novel Coronaviruses[Title/Abstract])) OR (2019-nCoV[Title/Abstract])) OR (Coronavirus 2, SARS[Title/Abstract])) OR (Coronavirus Disease 19[Title/Abstract])) OR (Coronavirus Disease 2019[Title/Abstract])) OR (Coronavirus Disease-19[Title/Abstract])) OR (Coronavirus, 2019 Novel[Title/Abstract])) OR (Coronavirus, Wuhan[Title/Abstract])) OR (COVID 19[Title/Abstract])) OR (COVID19[Title/Abstract])) OR (Disease 2019, Coronavirus[Title/Abstract])) OR (Novel Coronavirus, 2019[Title/Abstract])) OR (SARS Coronavirus 2[Title/Abstract])) OR (SARS CoV 2 Infection[Title/Abstract])) OR (SARS CoV 2 Virus[Title/Abstract])) OR (SARS-CoV-2[Title/Abstract])) OR (Severe Acute Respiratory Syndrome Coronavirus 2[Title/Abstract])) OR (Wuhan Coronavirus[Title/Abstract])) OR (Wuhan Seafood Market Pneumonia Virus[Title/Abstract]))) AND (((Diabetes[Title/Abstract]) OR (Hyperglycemia[Title/Abstract])) OR (Hyperglycaemia[Title/Abstract]))) AND ((((((new onset[Title/Abstract]) OR (new-onset[Title/Abstract])) OR (incident[Title/Abstract])) OR (transient[Title/Abstract])) OR (secondary[Title/Abstract])) OR (newly diagnosed[Title/Abstract]))

**Search results: 716 items**

***Cochrane library***

| **ID** | **Search** | **Results** |
| --- | --- | --- |
| #1 | COVID-19 | 10051 |
| #2 | (2019 nCoV Disease):ti,ab,kw OR (2019 nCoV Infection):ti,ab,kw OR (2019 Novel Coronavirus):ti,ab,kw OR (2019 Novel Coronaviruses):ti,ab,kw OR (2019 nCoV):ti,ab,kw OR (Coronavirus 2, SARS):ti,ab,kw OR (Coronavirus Disease 19):ti,ab,kw OR (Coronavirus Disease 2019):ti,ab,kw OR (Coronavirus Disease-19):ti,ab,kw OR (Coronavirus, 2019 Novel):ti,ab,kw OR (Coronavirus, Wuhan):ti,ab,kw OR (COVID 19):ti,ab,kw OR (COVID19):ti,ab,kw OR (Disease 2019, Coronavirus):ti,ab,kw OR (Novel Coronavirus, 2019):ti,ab,kw OR (SARS Coronavirus 2):ti,ab,kw OR (SARS CoV 2 Infection):ti,ab,kw OR (SARS CoV 2 Virus):ti,ab,kw OR (SARS-CoV-2):ti,ab,kw OR (Severe Acute Respiratory Syndrome Coronavirus 2):ti,ab,kw OR (Wuhan Coronavirus):ti,ab,kw OR (Wuhan Seafood Market Pneumonia Virus):ti,ab,kw | 10287 |
| #3 | #1 or #2 | 10495 |
| #4 | Diabetes | 95722 |
| #5 | (Hyperglycemia):ti,ab,kw OR (Hyperglycaemia):ti,ab,kw | 9096 |
| #6 | #4 or #5 | 99261 |
| #7 | new onset | 11803 |
| #8 | (new-onset):ti,ab,kw OR (incident):ti,ab,kw OR (transient):ti,ab,kw OR (secondary):ti,ab,kw OR (newly diagnosed):ti,ab,kw | 330901 |
| #9 | #7 or #8 | 337063 |
| #10 | #3 and #6 and #9 | 252 |

**Search results: 252 items**

***Web of Science***

| **Set** | **Results** | **Search history** |
| --- | --- | --- |
| #4 | 1,137 | #3 AND #2 AND #1 |
| #3 | 4,458,095 | TS=(new onset or new-onset or incident or transient or secondary or newly diagnosed) |
| #2 | 1,245,466 | TS=(Diabetes or Hyperglycemia or Hyperglycaemia) |
| #1 | 359,329 | TS=(COVID-19 or 2019 nCoV Disease or 2019 nCoV Infection or 2019 Novel Coronavirus or 2019 Novel Coronaviruses or 2019-nCoV or Coronavirus 2, SARS or Coronavirus Disease 19 or Coronavirus Disease 2019 or Coronavirus Disease-19 or Coronavirus, 2019 Novel or Coronavirus, Wuhan or COVID 19 or COVID19 or Disease 2019, Coronavirus or Novel Coronavirus, 2019 or SARS Coronavirus 2 or SARS CoV 2 Infection or SARS CoV 2 Virus or SARS-CoV-2 or Severe Acute Respiratory Syndrome Coronavirus 2 or Wuhan Coronavirus or Wuhan Seafood Market Pneumonia Virus) |

**Search results: 1137 items**

***Embase***

| **No.** | **Query** | **Results** |
| --- | --- | --- |
| #4 | #1 AND #2 AND #3 | 1149 |
| #3 | 'new onset':ab,ti OR 'new-onset':ab,ti OR 'incident':ab,ti OR 'transient':ab,ti OR 'secondary':ab,ti OR 'newly diagnosed':ab,ti | 1701635 |
| #2 | 'diabetes':ab,ti OR 'hyperglycemia':ab,ti OR 'hyperglycaemia':ab,ti | 944406 |
| #1 | 'covid-19':ab,ti OR '2019 ncov disease':ab,ti OR '2019 ncov infection':ab,ti OR '2019 novel coronavirus':ab,ti OR '2019 novel coronaviruses':ab,ti OR '2019-ncov':ab,ti OR 'coronavirus 2, sars':ab,ti OR 'coronavirus disease 19':ab,ti OR 'coronavirus disease 2019':ab,ti OR 'coronavirus disease-19':ab,ti OR 'coronavirus, 2019 novel':ab,ti OR 'coronavirus, wuhan':ab,ti OR 'covid 19':ab,ti OR 'covid19':ab,ti OR 'disease 2019, coronavirus':ab,ti OR 'novel coronavirus, 2019':ab,ti OR 'sars coronavirus 2':ab,ti OR 'sars cov 2 infection':ab,ti OR 'sars cov 2 virus':ab,ti OR 'sars-cov-2':ab,ti OR 'severe acute respiratory syndrome coronavirus 2':ab,ti OR 'wuhan coronavirus':ab,ti OR 'wuhan seafood market pneumonia virus':ab,ti | 245848 |

**Search results: 1149 items**

**2. Excluded articles at full-text assessment (n=84)**

| **S/N** | **First author and year** | **Reason of exclusion** |
| --- | --- | --- |
| 1 | Al-Abdulrazzaq, et al. 2022 (1) | Subject not relevant |
| 2 | Alaqeel, et al. 2021 (2) | Subject not relevant |
| 3 | Alfishawy, et al. 2021 (3) | Case Reports |
| 4 | Ambati, et al. 2022 (4) | Case Reports |
| 5 | Ata, et al. 2022 (5) | Subject not relevant |
| 6 | Bhatti, et al. 2020 (6) | Subject not relevant |
| 7 | Bornstein, et al. 2021 (7) | Subject not relevant |
| 8 | Botelho, et al. 2022 (8) | Subject not relevant |
| 9 | Bu, et al. 2022 (9) | Subject not relevant |
| 10 | Cariou, et al. 2021 (10) | Subject not relevant |
| 11 | Catriona, et al. 2022 (11) | Letter to the editor |
| 12 | Chao, et al. 2021 (12) | Subject not relevant |
| 13 | Chao, et al. 2022 (13) | Subject not relevant |
| 14 | Chen, et al. 2021 (14) | Data unavailable |
| 15 | Das, et al. 2021 (15) | Data unavailable |
| 16 | Dilek, et al. 2021 (16) | Case Reports |
| 17 | Donbaloğlu, et al. 2022 (17) | Case Reports |
| 18 | Elbarbary, et al. 2020 (18) | Subject not relevant |
| 19 | Elbarbary, et al. 2021 (19) | Subject not relevant |
| 20 | Ghosh, et al. 2021 (20) | Subject not relevant |
| 21 | Goldman, et al. 2022 (21) | Data unavailable |
| 22 | Gómez, et al. 2021 (22) | Subject not relevant |
| 23 | Gottesman, et al. 2022 (23) | Subject not relevant |
| 24 | Hasanzad, et al. 2022 (24) | Letter to the editor |
| 25 | Heaney, et al. 2020 (25) | Case Reports |
| 26 | Ho, et al. 2021 (26) | Subject not relevant |
| 27 | Jacob, et al. 2021 (27) | Subject not relevant |
| 28 | Jia, et al. 2021 (28) | Subject not relevant |
| 29 | Kaestner, et al. 2021 (29) | Case Reports |
| 30 | Kamrath, et al. 2021 (30) | Subject not relevant |
| 31 | Kamrath, et al. 2022 (31) | Subject not relevant |
| 32 | Kaya, et al. 2022 (32) | Subject not relevant |
| 33 | Khunti, et al. 2021 (33) | Meta-Analysis |
| 34 | Kostopoulou, et al. 2021 (34) | Subject not relevant |
| 35 | Koufakis, et al. 2021 (35) | Subject not relevant |
| 36 | Kountouri, et al. 2021 (36) | Review |
| 37 | Kwiendacz, et al. 2021 (37) | Case Reports |
| 38 | Lawrence, et al. 2021 (38) | Subject not relevant |
| 39 | Lazzeroni, et al. 2020 (39) | Case Reports |
| 40 | Li, et al. 2021 (40) | Subject not relevant |
| 41 | Luciano, et al. 2022 (41) | Case Reports |
| 42 | Lui, et al. 2022 (42) | Subject not relevant |
| 43 | Makker, et al. 2021 (43) | Subject not relevant |
| 44 | Mameli, et al. 2021 (44) | Subject not relevant |
| 45 | Marchand, et al. 2020 (45) | Case Reports |
| 46 | Mastromauro, et al. 2022 (46) | Data unavailable |
| 47 | McGlacken-Byrne, et al. 2021 (47) | Data unavailable |
| 48 | Mendes, et al. 2022 (48) | Review |
| 49 | Messaaoui, et al. 2021 (49) | Subject not relevant |
| 50 | Misra, et al. 2021 (50) | Subject not relevant |
| 51 | Mistry, et al. 2021 (51) | Data unavailable |
| 52 | Modarelli, et al. 2022 (52) | Subject not relevant |
| 53 | Nagl, et al. 2022 (53) | Subject not relevant |
| 54 | Nair, et al. 2022 (54) | Subject not relevant |
| 55 | Nguyen, et al. 2021 (55) | Subject not relevant |
| 56 | Nóvoa-Medina, et al. 2022 (56) | Subject not relevant |
| 57 | Orioli, et al. 2021 (57) | Data unavailable |
| 58 | Patel, et al. 2021 (58) | Subject not relevant |
| 59 | Pick, et al. 2021 (59) | Case Reports |
| 60 | Rabbone, et al. 2020 (60) | Subject not relevant |
| 61 | Rathmann, et al. (61) | Data unavailable |
| 62 | Rebello, et al. 2022 (62) | Subject not relevant |
| 63 | Rubino, et al. 2020 (63) | Letter to the editor |
| 64 | Rusak, et al. 2022 (64) | Subject not relevant |
| 65 | Sachdeva, et al. 2020 (65) | Meta-Analysis |
| 66 | Salmi, et al. 2022 (66) | Subject not relevant |
| 67 | Samuel, et al. 2021 (67) | Review |
| 68 | Samuel, et al. 2022 (68) | Subject not relevant |
| 69 | Sathish 2022 (69) | Letter to the editor |
| 70 | Sathish, et al. 2021 (70) | Data unavailable |
| 71 | Sathish, et al. 2021 (71) | Meta-Analysis |
| 72 | Sathish, et al. 2021 (72) | Letter to the editor |
| 73 | Schmitt, et al. 2022 (73) | Subject not relevant |
| 74 | Shawar, et al. 2021 (74) | Case Reports |
| 75 | Sherif, et al. 2020 (75) | Subject not relevant |
| 76 | Sherif, et al. 2021 (76) | Subject not relevant |
| 77 | Singh, et al. 2022 (77) | Review |
| 78 | Sourij, et al. 2021 (78) | Subject not relevant |
| 79 | Uchihara, et al. 2022 (79) | Subject not relevant |
| 80 | Vlad, et al. 2021 (80) | Subject not relevant |
| 81 | Wang, et al. 2021 (81) | Review |
| 82 | Wolf, et al. (82) | Subject not relevant |
| 83 | Wu, et al. 2020 (83) | Subject not relevant |
| 84 | Yonekawa, et al. 2022 (84) | Review |

**Reference:**

1. Al-Abdulrazzaq D, Alkandari A, Alhusaini F, et al. Higher rates of diabetic ketoacidosis and admission to the paediatric intensive care unit among newly diagnosed children with type 1 diabetes in Kuwait during the COVID-19 pandemic. *DIABETES-METABOLISM RESEARCH AND REVIEWS.* 2022;38(3).
2. Alaqeel A, Aljuraibah F, Alsuhaibani M, et al. The Impact of COVID-19 Pandemic Lockdown on the Incidence of New-Onset Type 1 Diabetes and Ketoacidosis Among Saudi Children. *Frontiers in Endocrinology.* 2021;12.
3. Alfishawy M, Nassar M, Mohamed M, Fatthy M, Elmessiery RM. New-onset Type 1 Diabetes Mellitus with Diabetic Ketoacidosis and Pancreatitis in a Patient with COVID-19. *Sci Afr.* 2021;13:e00915.
4. Ambati S, Mihic M, Rosario DC, Sanchez J, Bakar A. New-Onset Type 1 Diabetes in Children With SARS-CoV-2 Infection. *Cureus.* 2022;14(3):e22790.
5. Ata A, Jalilova A, Kırkgöz T, et al. Does COVID-19 predispose patients to type 1 diabetes mellitus? *Clin Pediatr Endocrinol.* 2022;31(1):33-37.
6. Bhatti R, Khamis AH, Khatib S, Shiraz S, Matfin G. Clinical Characteristics and Outcomes of Patients With Diabetes Admitted for COVID-19 Treatment in Dubai: Single-Centre Cross-Sectional Study. *JMIR Public Health Surveill.* 2020;6(4):e22471.
7. Bornstein SR, Guan K, Brunßen C, et al. The transCampus Metabolic Training Programme Explores the Link of SARS-CoV-2 Virus to Metabolic Disease. *Horm Metab Res.* 2021;53(3):204-206.
8. Botelho TA, Santos JMN, Pinho CMS, et al. Ketoacidosis in new-onset type 1 diabetes: did the severity increase during the COVID-19 pandemic? *JOURNAL OF PEDIATRIC ENDOCRINOLOGY & METABOLISM.* 2022;35(1):73-77.
9. Bu W, E L, Q C, et al. Imaging of the Pancreas (IMAP) in New-Onset Diabetes (NOD): A Prospective Pilot Study. *Clin Transl Gastroenterol.* 2022.
10. Cariou B, Pichelin M, Goronflot T, et al. Phenotypic characteristics and prognosis of newly diagnosed diabetes in hospitalized patients with COVID-19: Results from the CORONADO study. *Diabetes Research and Clinical Practice.* 2021;175.
11. Catriona C, Paolo P. SARS-CoV-2 induced post-translational protein modifications: A trigger for developing autoimmune diabetes? *Diabetes Metab Res Rev.* 2022;38(1):e3508.
12. Chao JY, Sugarman A, Kimura A, et al. Factors Associated With Hospitalization in Children and Adolescents With SARS-CoV-2 Infection. *Clin Pediatr (Phila).* 2022;61(2):159-167.
13. Chao LC, Vidmar AP, Georgia S. Spike in diabetic ketoacidosis rates in pediatric type 2 diabetes during the COVID-19 pandemic. *Diabetes Care.* 2021;44(6):1451-1453.
14. Chen M, Zhu B, Chen D, et al. COVID-19 May Increase the Risk of Insulin Resistance in Adult Patients Without Diabetes: A 6-Month Prospective Study. *Endocrine Practice.* 2021;27(8):834-841.
15. Das L, Bhadada SK. COVID-19-associated new-onset hyperglycaemia: A reversible entity or persistent risk? *Postgraduate Medical Journal.* 2021.
16. Dilek SÖ, Gürbüz F, Turan I, Celiloǧlu C, Yüksel B. Changes in the presentation of newly diagnosed type 1 diabetes in children during the COVID-19 pandemic in a tertiary center in Southern Turkey. *Journal of Pediatric Endocrinology and Metabolism.* 2021;34(10):1303-1309.
17. Donbaloğlu Z, Tuhan H, Tural Kara T, et al. The Examination of the Relationship Between COVID-19 and New-Onset Type 1 Diabetes Mellitus in Children. *Turk Arch Pediatr.* 2022;57(2):222-227.
18. Elbarbary NS, dos Santos TJ, de Beaufort C, Agwu JC, Calliari LE, Scaramuzza AE. COVID-19 outbreak and pediatric diabetes: Perceptions of health care professionals worldwide. *Pediatric Diabetes.* 2020;21(7):1083-1092.
19. Elbarbary NS, Dos Santos TJ, de Beaufort C, Wiltshire E, Pulungan A, Scaramuzza AE. The Challenges of Managing Pediatric Diabetes and Other Endocrine Disorders During the COVID-19 Pandemic: Results From an International Cross-Sectional Electronic Survey. *Front Endocrinol (Lausanne).* 2021;12:735554.
20. Ghosh A, Anjana RM, Shanthi Rani CS, et al. Glycemic parameters in patients with new-onset diabetes during COVID-19 pandemic are more severe than in patients with new-onset diabetes before the pandemic: NOD COVID India Study. *Diabetes and Metabolic Syndrome: Clinical Research and Reviews.* 2021;15(1):215-220.
21. Goldman S, Pinhas-Hamiel O, Weinberg A, et al. Alarming increase in ketoacidosis in children and adolescents with newly diagnosed type 1 diabetes during the first wave of the COVID-19 pandemic in Israel. *Pediatr Diabetes.* 2022;23(1):10-18.
22. Gómez AM, Henao DC, Muñoz OM, et al. Glycemic control metrics using flash glucose monitoring and hospital complications in patients with COVID-19. *Diabetes and Metabolic Syndrome: Clinical Research and Reviews.* 2021;15(2):499-503.
23. Gottesman BL, Yu J, Tanaka C, Longhurst CA, Kim JJ. Incidence of New-Onset Type 1 Diabetes Among US Children During the COVID-19 Global Pandemic. *JAMA Pediatr.* 2022;176(4):414-415.
24. Hasanzad M, Larijani B, Aghaei Meybodi HR. Diabetes and COVID-19: a bitter nightmare. *J Diabetes Metab Disord.* 2022:1-3.
25. Heaney AI, Griffin GD, Simon EL. Newly diagnosed diabetes and diabetic ketoacidosis precipitated by COVID-19 infection. *American Journal of Emergency Medicine.* 2020;38(11):2491.e2493-2491.e2494.
26. Ho J, Rosolowsky E, Pacaud D, et al. Diabetic ketoacidosis at type 1 diabetes diagnosis in children during the COVID-19 pandemic. *PEDIATRIC DIABETES.* 2021;22(4):552-557.
27. Jacob R, Weiser G, Krupik D, et al. Diabetic Ketoacidosis at Emergency Department Presentation During the First Months of the SARS-CoV-2 Pandemic in Israel: A Multicenter Cross-Sectional Study. *Diabetes Therapy.* 2021;12(5):1569-1574.
28. Jia X, Gesualdo P, Geno Rasmussen C, et al. Prevalence of SARS-CoV-2 Antibodies in Children and Adults with Type 1 Diabetes. *Diabetes Technology and Therapeutics.* 2021;23(7):517-521.
29. Kaestner R, Harsch IA. COVID-19 infection as a trigger for new-onset type 1 diabetes in a susceptible individual - or just coincidence? *GMS HYGIENE AND INFECTION CONTROL.* 2021;16.
30. Kamrath C, Rosenbauer J, Eckert AJ, et al. Incidence of COVID-19 and Risk of Diabetic Ketoacidosis in New-Onset Type 1 Diabetes. *PEDIATRICS.* 2021;148(3).
31. Kamrath C, Rosenbauer J, Eckert AJ, et al. Incidence of Type 1 Diabetes in Children and Adolescents During the COVID-19 Pandemic in Germany: Results From the DPV Registry. *Diabetes Care.* 2022.
32. Kaya G, Cimbek EA, Yeşilbaş O, Bostan YE, Karagüzel G. A Long-Term Comparison of Presenting Characteristics of Children with Newly Diagnosed Type 1 Diabetes Before and During the COVID-19 Pandemic. *J Clin Res Pediatr Endocrinol.* 2022.
33. Khunti K, Del Prato S, Mathieu C, Kahn SE, Gabbay RA, Buse JB. COVID-19, Hyperglycemia, and New-Onset Diabetes. *Diabetes Care.* 2021;44(12):2645-2655.
34. Kostopoulou E, Eliopoulou MI, Gil APR, Chrysis D. Impact of COVID-19 on new-onset type 1 diabetes mellitus - A one-year prospective study. *EUROPEAN REVIEW FOR MEDICAL AND PHARMACOLOGICAL SCIENCES.* 2021;25(19):5928-5935.
35. Koufakis T, Metallidis S, Zebekakis P, Kotsa K. Intestinal SGLT1 as a therapeutic target in COVID-19-related diabetes: A "two-edged sword" hypothesis. *Br J Clin Pharmacol.* 2021;87(10):3643-3646.
36. Kountouri A, Korakas E, Ikonomidis I, et al. Type 1 Diabetes Mellitus in the SARS-CoV-2 Pandemic: Oxidative Stress as a Major Pathophysiological Mechanism Linked to Adverse Clinical Outcomes. *Antioxidants (Basel).* 2021;10(5).
37. Kwiendacz H, Nabrdalik K, Dobrakowski M, Gumprecht J. Asymptomatic COVID-19 mimicking disseminated carcinoma in a patient with new-onset type 2 diabetes. *Pol Arch Intern Med.* 2021;131(10).
38. Lawrence C, Seckold R, Smart C, et al. Increased paediatric presentations of severe diabetic ketoacidosis in an Australian tertiary centre during the COVID-19 pandemic. *Diabetic Medicine.* 2021;38(1).
39. Lazzeroni P, Bernardi L, Pecora F, et al. Diabetic ketoacidosis at type 1 diabetes onset: Indirect impact of covid-19 pandemic. *Acta Biomedica.* 2020;91(4):1-5.
40. Li Y, Han X, Huang J, et al. Follow-up study of pulmonary sequelae in discharged COVID-19 patients with diabetes or secondary hyperglycemia. *Eur J Radiol.* 2021;144:109997.
41. Luciano TM, Halah MP, Sarti MTA, et al. DKA and new-onset type 1 diabetes in Brazilian children and adolescents during the COVID-19 pandemic. *Arch Endocrinol Metab.* 2022;66(1):88-91.
42. Lui DTW, Lee CH, Tan KCB. One year into the clash of pandemics of diabetes and COVID-19: Lessons learnt and future perspectives. *J Diabetes Investig.* 2022;13(1):19-21.
43. Makker J, Sun H, Patel H, et al. Impact of Prediabetes and Type-2 Diabetes on Outcomes in Patients with COVID-19. *International Journal of Endocrinology.* 2021;2021.
44. Mameli C, Scaramuzza A, Macedoni M, et al. Type 1 diabetes onset in Lombardy region, Italy, during the COVID-19 pandemic: The double-wave occurrence. *EClinicalMedicine.* 2021;39:101067.
45. Marchand L, Pecquet M, Luyton C. Type 1 diabetes onset triggered by COVID-19. *ACTA DIABETOLOGICA.* 2020;57(10):1265-1266.
46. Mastromauro C, Blasetti A, Primavera M, et al. Peculiar characteristics of new-onset Type 1 Diabetes during COVID-19 pandemic. *Ital J Pediatr.* 2022;48(1):26.
47. McGlacken-Byrne SM, Drew SEV, Turner K, Peters C, Amin R. The SARS-CoV-2 pandemic is associated with increased severity of presentation of childhood onset type 1 diabetes mellitus: A multi-centre study of the first COVID-19 wave. *Diabet Med.* 2021;38(9):e14640.
48. Mendes TB, Câmara-de-Souza AB, Halpern B. Hospital management of hyperglycemia in the context of COVID-19: evidence-based clinical considerations. *Diabetol Metab Syndr.* 2022;14(1):37.
49. Messaaoui A, Hajselova L, Tenoutasse S. Anti-SARS-CoV-2 antibodies in new-onset type 1 diabetes in children during pandemic in Belgium. *J Pediatr Endocrinol Metab.* 2021;34(10):1319-1322.
50. Misra S, Barron E, Vamos E, et al. Temporal trends in emergency admissions for diabetic ketoacidosis in people with diabetes in England before and during the COVID-19 pandemic: a population-based study. *Lancet Diabetes Endocrinol.* 2021;9(10):671-680.
51. Mistry S, Gouripeddi R, Facelli JC. Data-driven identification of temporal glucose patterns in a large cohort of nondiabetic patients with COVID-19 using time-series clustering. *JAMIA OPEN.* 2021;4(3).
52. Modarelli R, Sarah S, Ramaker ME, Bolobiongo M, Benjamin R, Gumus Balikcioglu P. Pediatric Diabetes on the Rise: Trends in Incident Diabetes During the COVID-19 Pandemic. *J Endocr Soc.* 2022;6(4):bvac024.
53. Nagl K, Waldhoer T, Hofer SE, et al. Alarming Increase of Ketoacidosis Prevalence at Type 1 Diabetes-Onset in Austria-Results From a Nationwide Registry. *FRONTIERS IN PEDIATRICS.* 2022;10.
54. Nair AM, Gopalan S, Rajendran V, et al. Role of secondary sepsis in COVID-19 mortality: Observations on patients with preexisting diabetes mellitus and newly diagnosed hyperglycemia. *Monaldi Arch Chest Dis.* 2022.
55. Nguyen NTV, Chau HN, Le NH, Nguyen HH, Nguyen HA. Impact of Novel Guidelines on Multifactorial Control and Its Association with Mortality in Adult Patients with Hypertension and Newly Diagnosed Type 2 Diabetes: A 4-Year Prospective Multicenter Study. *International Journal of Endocrinology.* 2021;2021.
56. Nóvoa-Medina Y, Pavlovic-Nesic S, González-Martín JM, et al. Role of the SARS-CoV-2 virus in the appearance of new onset type 1 diabetes mellitus in children in Gran Canaria, Spain. *J Pediatr Endocrinol Metab.* 2022;35(3):393-397.
57. Orioli L, Servais T, Belkhir L, et al. Clinical characteristics and short-term prognosis of in-patients with diabetes and COVID-19: A retrospective study from an academic center in Belgium. *Diabetes and Metabolic Syndrome: Clinical Research and Reviews.* 2021;15(1):149-157.
58. Patel U, Deluxe L, Salama C, et al. Evaluation of Characteristics and Outcomes for Patients with Diabetic Ketoacidosis (DKA) With and Without COVID-19 in Elmhurst Queens During Similar Three-Month Periods in 2019 and 2020. *Cureus.* 2021;13(7):e16427.
59. Pick AJ, Joyce M. Telehealth to Avoid Emergency Department Visit and Hospitalization for a Person With Newly Diagnosed Type 1 Diabetes During the Coronavirus Disease 2019 Pandemic. *Clin Diabetes.* 2021;39(4):445-448.
60. Rabbone I, Schiaffini R, Cherubini V, Maffeis C, Scaramuzza A, Italian Soc Pediat E. Has COVID-19 Delayed the Diagnosis and Worsened the Presentation of Type 1 Diabetes in Children? *DIABETES CARE.* 2020;43(11):2870-2872.
61. Rathmann W, Kuss O, Kostev K. Incidence of newly diagnosed diabetes after Covid-19. *DIABETOLOGIA.*
62. Rebello CJ, Axelrod CL, Reynolds CF, 3rd, Greenway FL, Kirwan JP. Exercise as a Moderator of Persistent Neuroendocrine Symptoms of COVID-19. *Exerc Sport Sci Rev.* 2022;50(2):65-72.
63. Rubino F, Amiel SA, Zimmet P, et al. New-Onset Diabetes in Covid-19. *The New England journal of medicine.* 2020.
64. Rusak E, Seget S, Macherski M, Furgał N, Dyś P, Jarosz-Chobot P. Has the COVID-19 Pandemic Affected the Prevalence of Diabetic Ketoacidosis in Polish Children with Newly Diagnosed Type 1 Diabetes? An Example of the Largest Polish Pediatric Diabetes Center (Upper Silesia-Katowice, Poland). *Healthcare (Basel).* 2022;10(2).
65. Sachdeva S, Desai R, Gupta U, Prakash A, Jain A, Aggarwal A. Admission Hyperglycemia in Non-diabetics Predicts Mortality and Disease Severity in COVID-19: a Pooled Analysis and Meta-summary of Literature. *SN Comprehensive Clinical Medicine.* 2020;2(11):2161-2166.
66. Salmi H, Heinonen S, Hästbacka J, et al. New-onset type 1 diabetes in Finnish children during the COVID-19 pandemic. *Arch Dis Child.* 2022;107(2):180-185.
67. Samuel SM, Varghese E, Büsselberg D. Therapeutic Potential of Metformin in COVID-19: Reasoning for Its Protective Role. *Trends Microbiol.* 2021;29(10):894-907.
68. Samuel SM, Varghese E, Triggle CR, Büsselberg D. COVID-19 Vaccines and Hyperglycemia-Is There a Need for Postvaccination Surveillance? *Vaccines (Basel).* 2022;10(3).
69. Sathish T. Risk of mortality in COVID-19 patients with newly diagnosed and pre-existing diabetes. *Prim Care Diabetes.* 2022;16(1):214.
70. Sathish T, Anton MC, Sivakumar T. New-onset diabetes in "long COVID". *J Diabetes.* 2021;13(8):693-694.
71. Sathish T, Cao Y. Is newly diagnosed diabetes as frequent as preexisting diabetes in COVID-19 patients? *Diabetes and Metabolic Syndrome: Clinical Research and Reviews.* 2021;15(1):147-148.
72. Sathish T, Chandrika Anton M. Newly diagnosed diabetes in patients with mild to moderate COVID-19. *Diabetes and Metabolic Syndrome: Clinical Research and Reviews.* 2021;15(2):569-571.
73. Schmitt JA, Ashraf AP, Becker DJ, Sen B. Changes in Type 2 diabetes trends in Children and Adolescents during the COVID-19 Pandemic. *J Clin Endocrinol Metab.* 2022.
74. Shawar RS, Cymbaluk AL, Bell JJ, et al. Isolation and Education During a Pandemic: Novel Telehealth Approach to Family Education for a Child With New-Onset Type 1 Diabetes and Concomitant COVID-19. *Clin Diabetes.* 2021;39(1):124-127.
75. Sherif AE, McFadyen R, Boyd J, et al. Study protocol for resolution of organ injury in acute pancreatitis (RESORP): an observational prospective cohort study. *BMJ Open.* 2020;10(12):e040200.
76. Sherif EM, Elhenawy YI, Matter RM, Aly HH, Thabet RA, Fereig YA. Clinical characteristics and outcome of hospitalized children and adolescent patients with type 1 diabetes during the COVID-19 pandemic: Data from a single center surveillance study in Egypt. *Journal of Pediatric Endocrinology and Metabolism.* 2021;34(7):925-936.
77. Singh AK, Khunti K. COVID-19 and Diabetes. *Annu Rev Med.* 2022;73:129-147.
78. Sourij H, Aziz F, Bräuer A, et al. COVID-19 fatality prediction in people with diabetes and prediabetes using a simple score upon hospital admission. *Diabetes, Obesity and Metabolism.* 2021;23(2):589-598.
79. Uchihara M, Bouchi R, Kodani N, et al. Impact of newly diagnosed diabetes on coronavirus disease 2019 severity and hyperglycemia. *J Diabetes Investig.* 2022.
80. Vlad A, Serban V, Timar R, et al. Increased Incidence of Type 1 Diabetes during the COVID-19 Pandemic in Romanian Children. *Medicina (Kaunas).* 2021;57(9).
81. Wang IE, Cooper G, Mousa SA. Diagnostic Approaches for COVID-19 and Its Associated Complications. *Diagnostics (Basel).* 2021;11(11).
82. Wolf RM, Noor N, Izquierdo R, et al. Increase in newly diagnosed type 1 diabetes in youth during the COVID-19 pandemic in the United States: A multi-center analysis. *PEDIATRIC DIABETES.*
83. Wu L, Girgis CM, Cheung NW. COVID-19 and diabetes: Insulin requirements parallel illness severity in critically unwell patients. *Clinical Endocrinology.* 2020;93(4):390-393.
84. Yonekawa A, Shimono N. Clinical Significance of COVID-19 and Diabetes: In the Pandemic Situation of SARS-CoV-2 Variants including Omicron (B.1.1.529). *Biology (Basel).* 2022;11(3).

**3. Figure legends of the supplementary**

**Figure S1.** Forest plot of the incidence of new-onset diabetes.


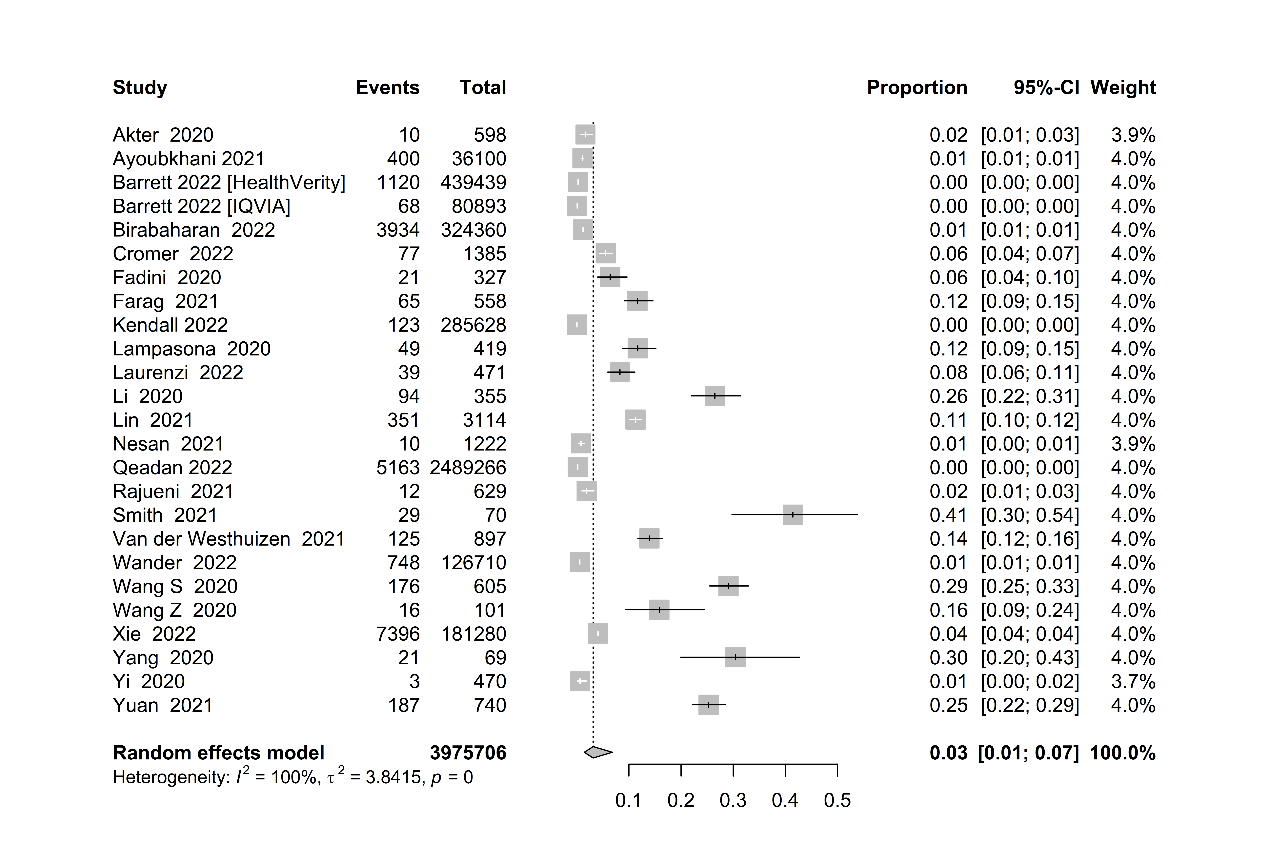


**Figure S2.** Forest plot of the incidence of new-onset hyperglycemia.


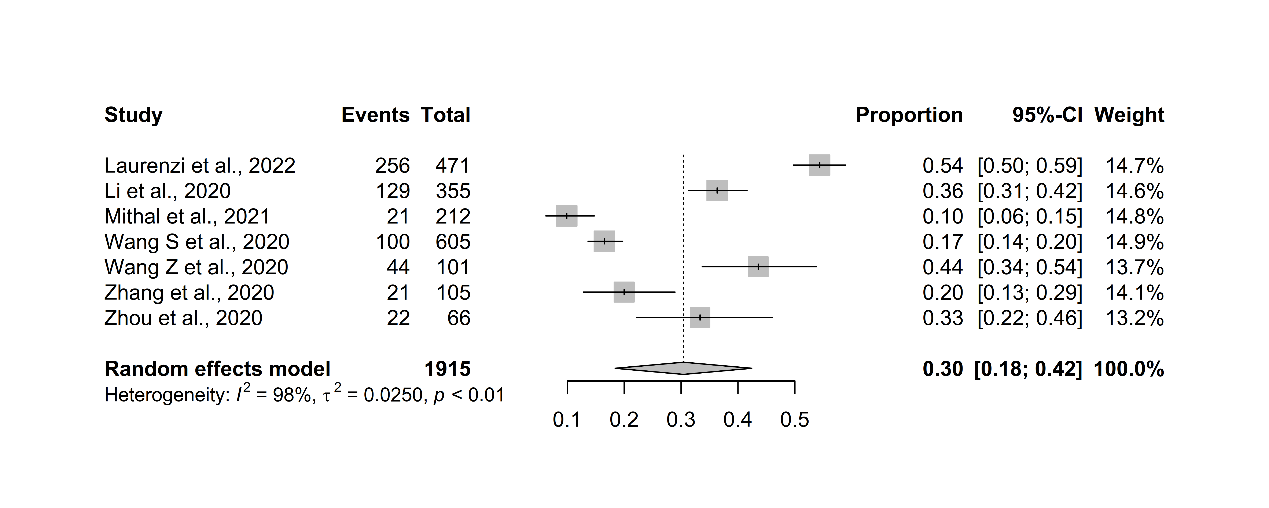


**Figure S3.** Forest plot of new-onset diabetes and hyperglycemia risk ratios.


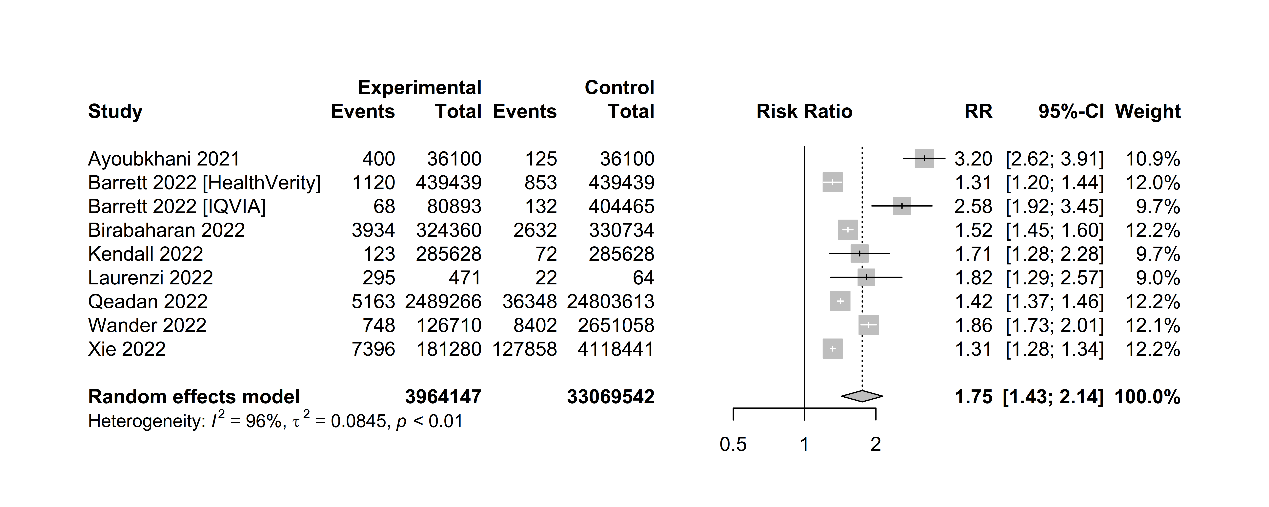


**Figure S4.** Percentage of men in the population with new-onset diabetes and hyperglycemia.


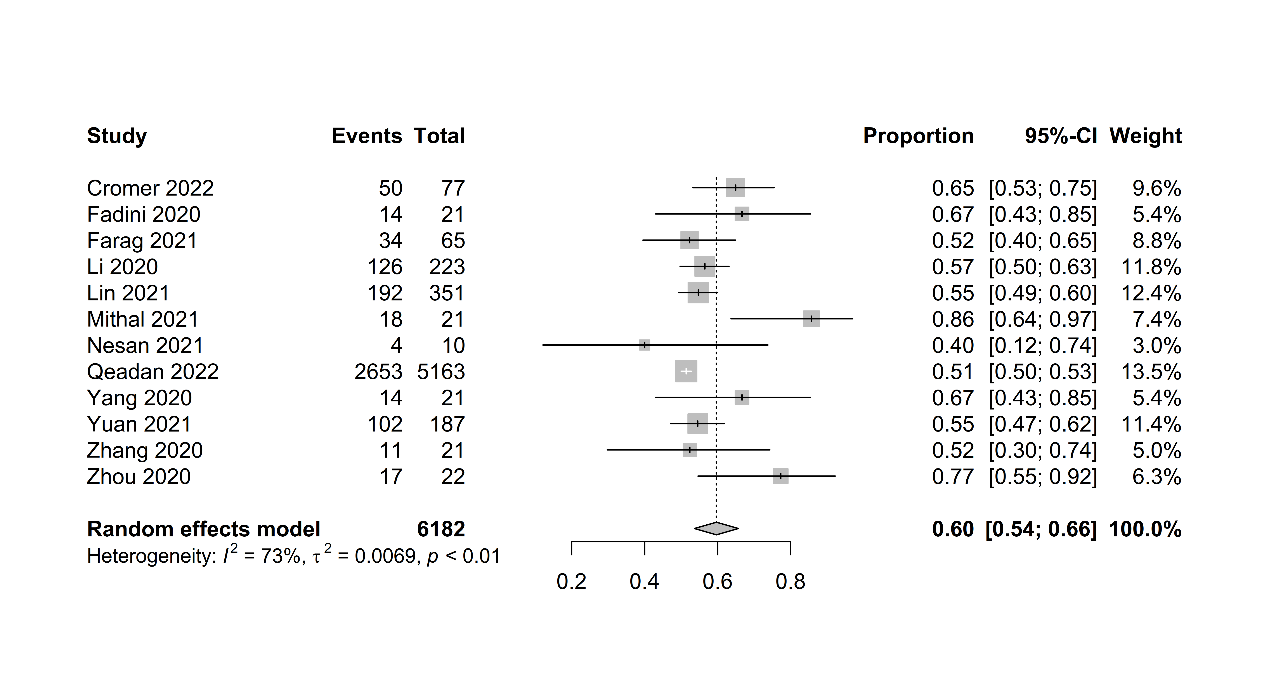


**Figure S5.** Percentage of women in the population with new-onset diabetes and hyperglycemia.


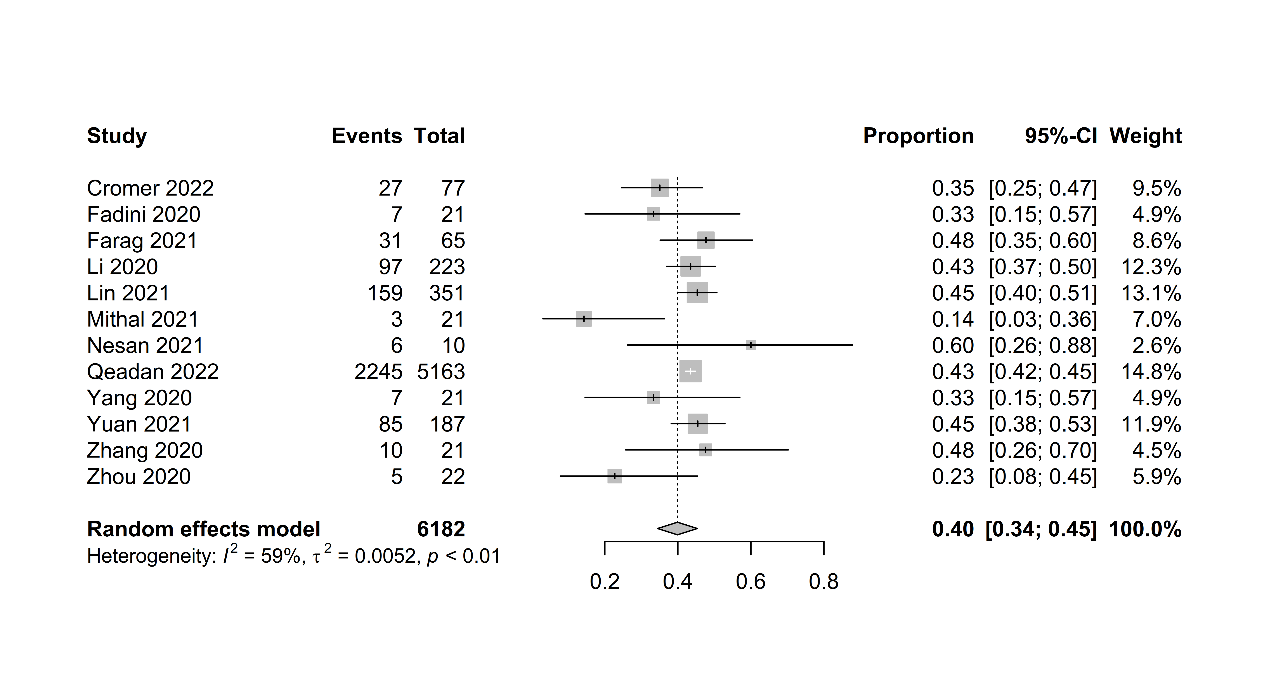


**Figure S6.** Forest plot of population mortality in patients with new-onset diabetes and hyperglycemia after COVID-19 positivity.


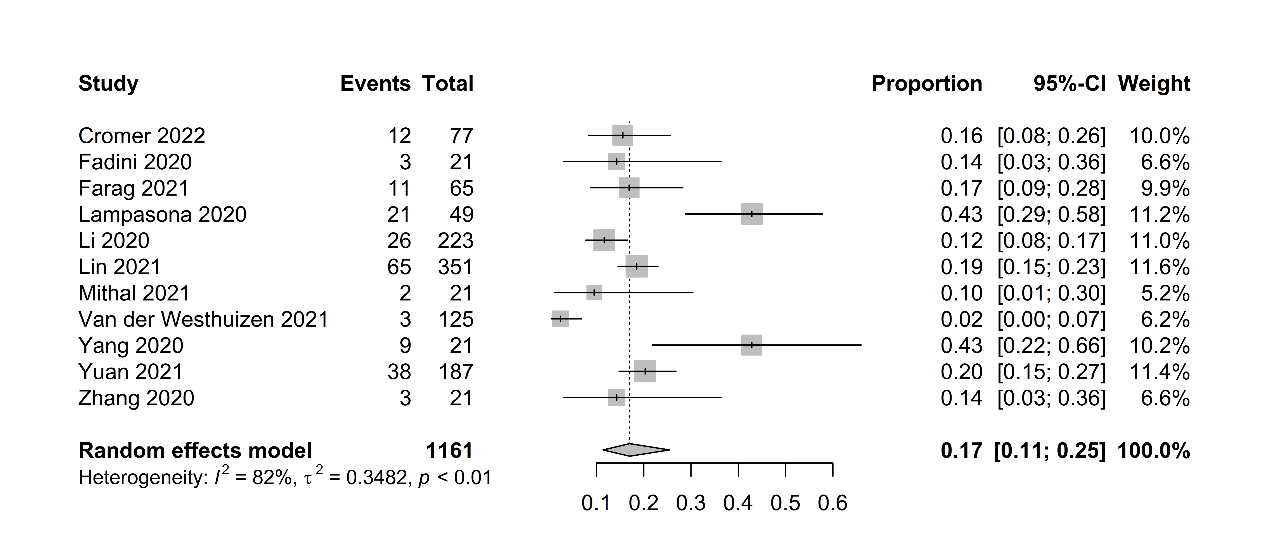


**Figure S7.** Forest plot of the incidence of new-onset diabetes and hyperglycemia in men after infection with COVID-19.


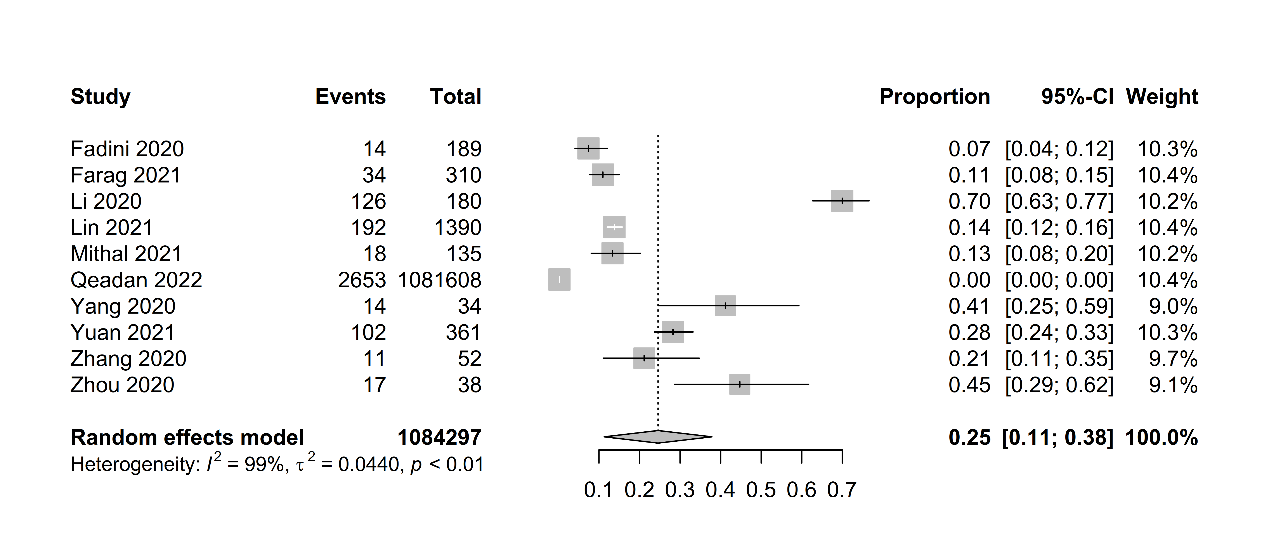


**Figure S8.** Forest plot of the incidence of new-onset diabetes and hyperglycemia in women infected with COVID-19.


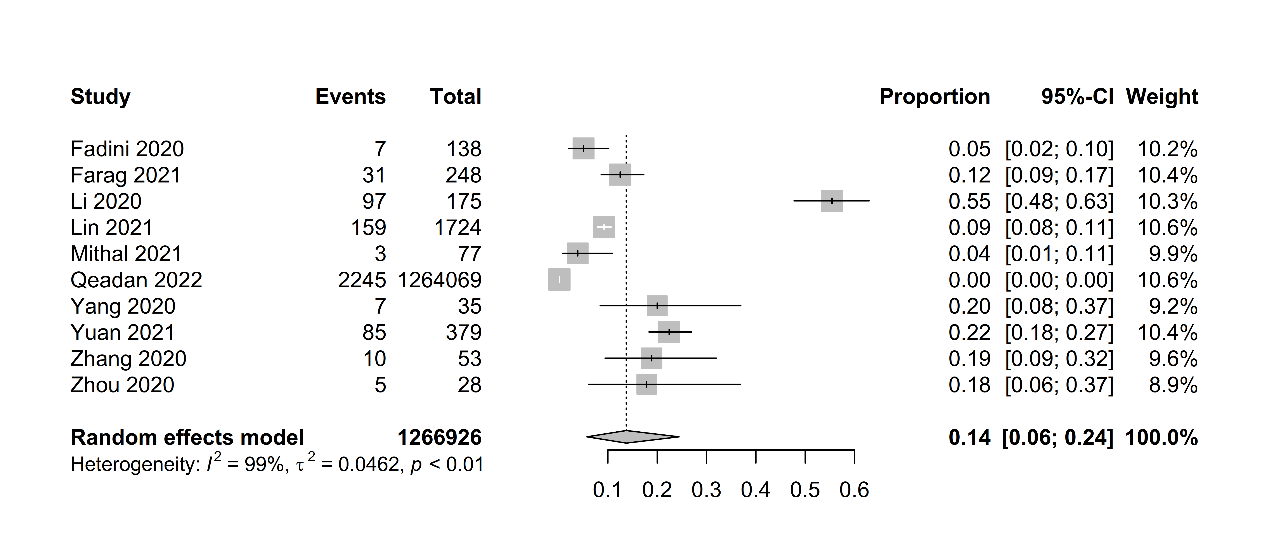


**Figure S9.** Forest plot for sensitivity analysis of the incidence of new-onset diabetes and hyperglycemia.


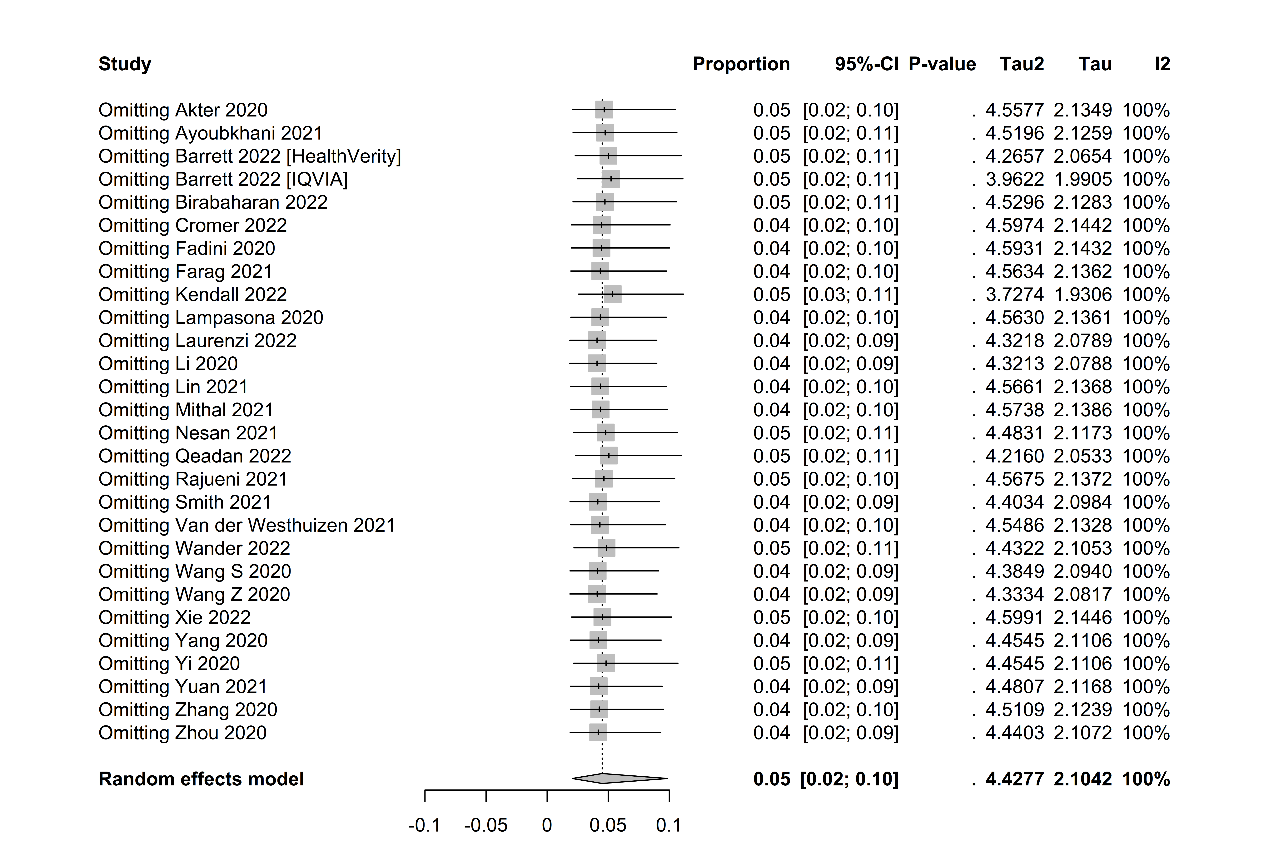


**Figure S10.** Forest plot for sensitivity analysis of the risk of developing new-onset diabetes and hyperglycemia.


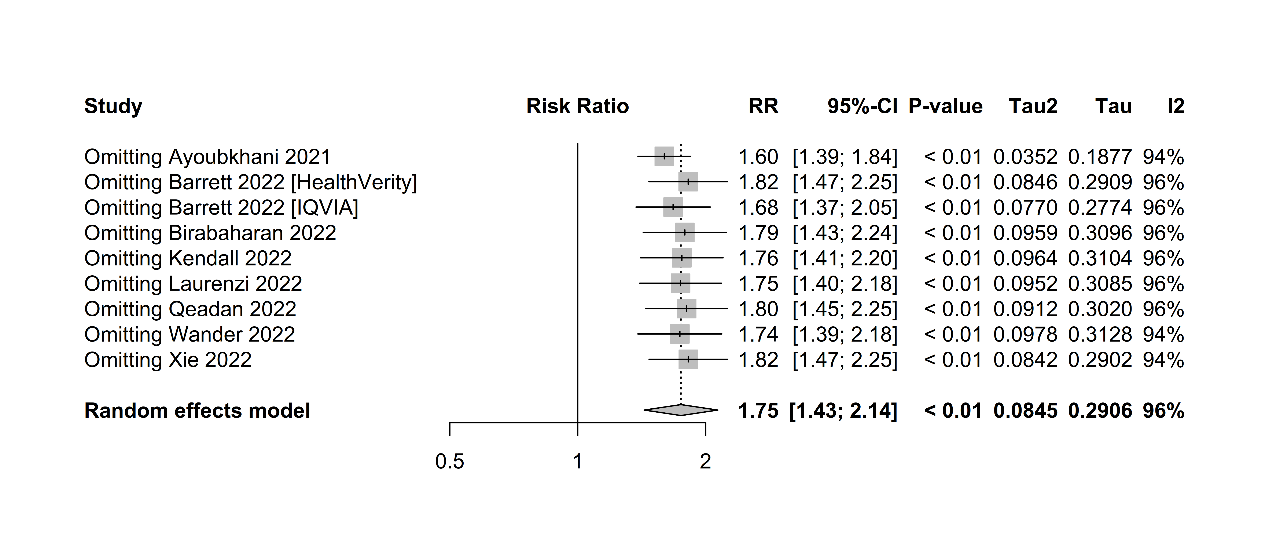


**Figure S11.** Forest plot for cumulative analysis of the incidence of new-onset diabetes and hyperglycemia.


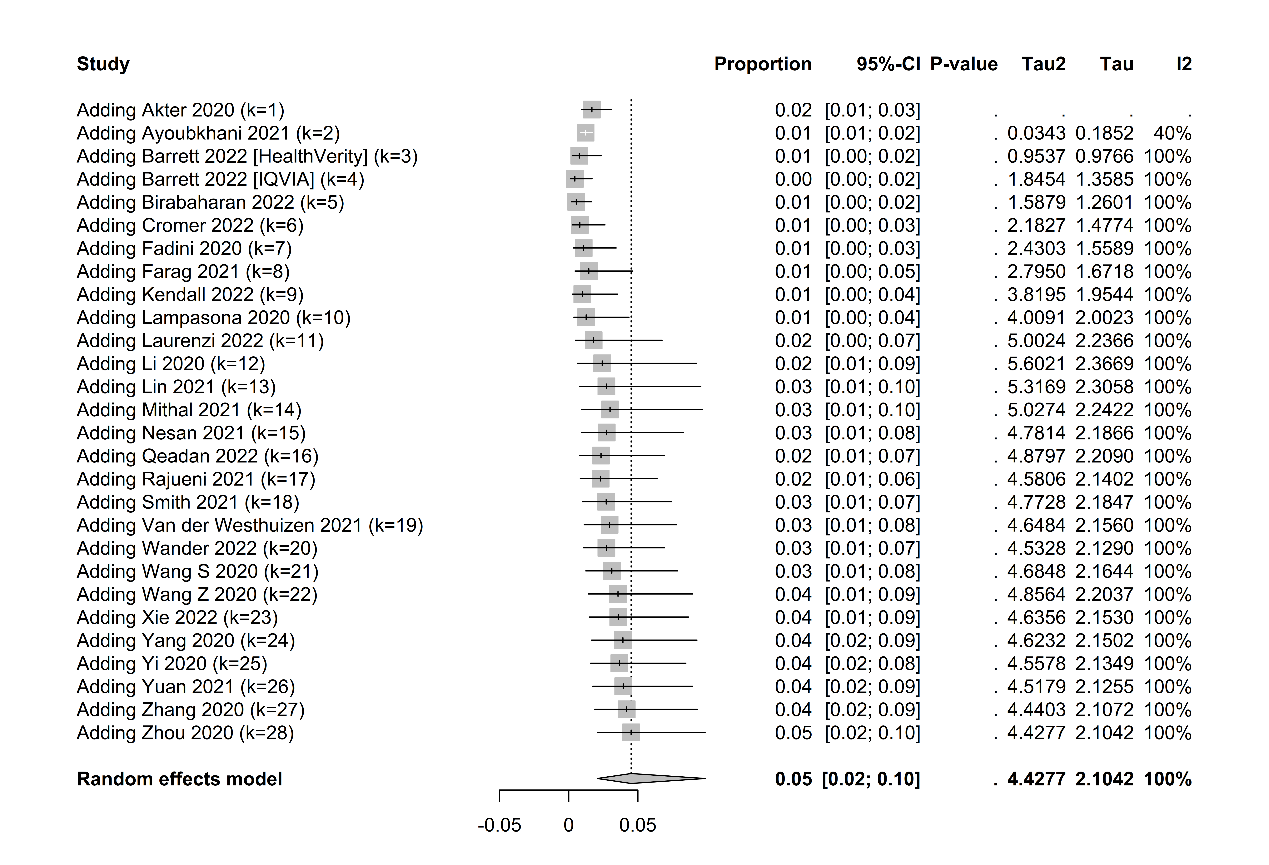


**Figure S12.** Forest plot for cumulative analysis of the risk of developing new-onset diabetes and hyperglycemia.


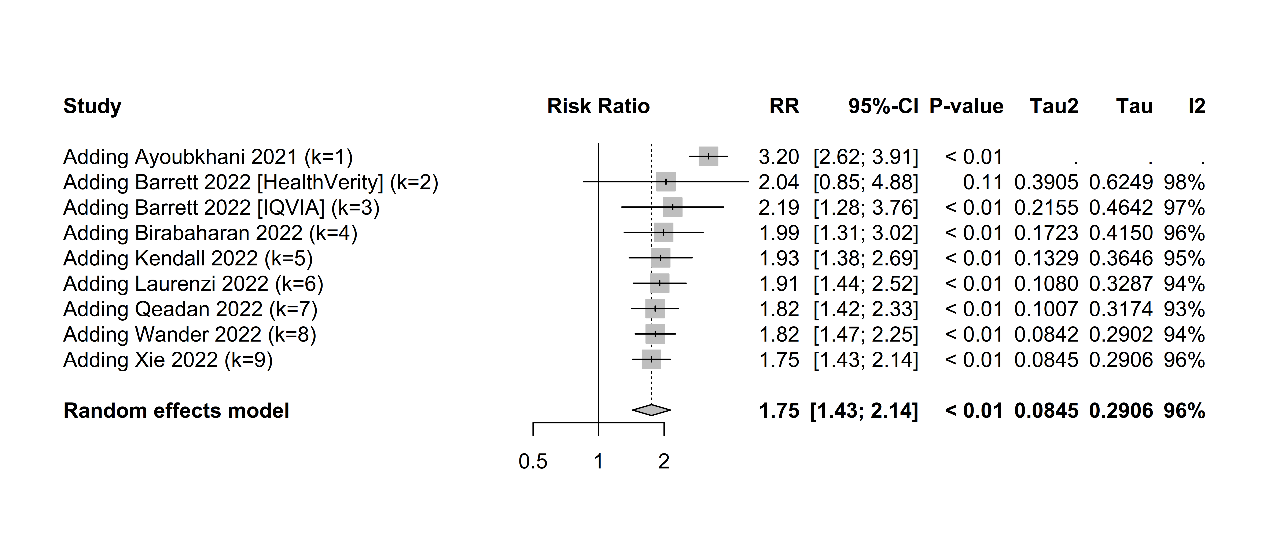


**Figure S13.** Funnel plot were performed to assess publication bias. A, Egger’s funnel plot. B, Begg’s funnel plot. C, General funnel plot. D, Adjusted funnel plot.


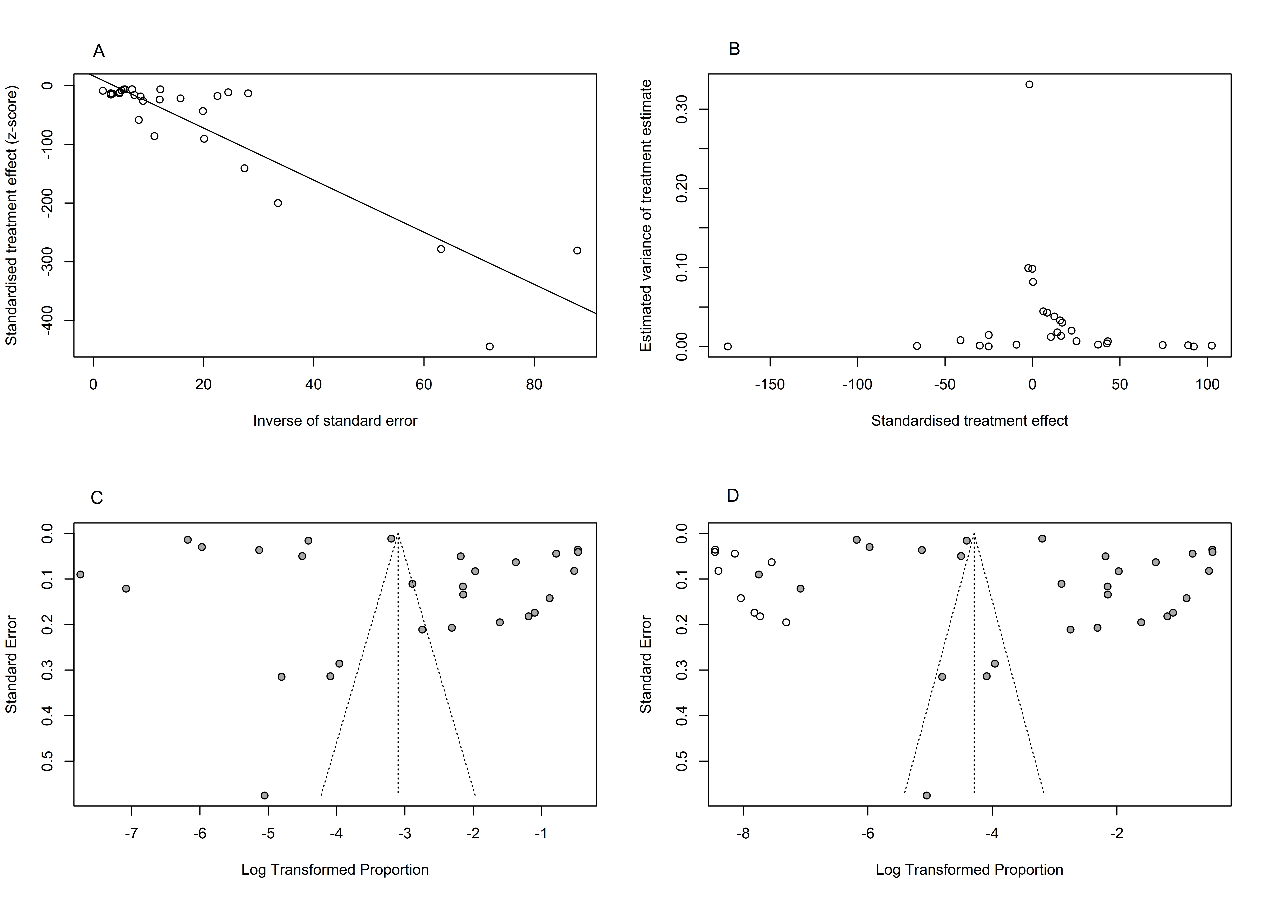

Supplement: Supplementary file 1 [file Data_Sheet_1.docx]
